# Exploring the Phytochemicals of *Acacia melanoxylon* R. Br.

**DOI:** 10.3390/plants10122698

**Published:** 2021-12-08

**Authors:** Diana Alves, Sidónio Duarte, Pedro Arsénio, Joana Gonçalves, Cecília M. P. Rodrigues, Ana Lourenço, Patrícia Máximo

**Affiliations:** 1 LAQV-REQUIMTE, Department of Chemistry, NOVA School of Science and Technology, NOVA University, 2829-516 Caparica, Portugal; dfl.alves@campus.fct.unl.pt (D.A.); sidonioduarte88@gmail.com (S.D.); 2 Linking Landscape, Environment, Agriculture and Food (LEAF)—Research Center, Associated Laboratory TERRA, Instituto Superior de Agronomia, Universidade de Lisboa, Tapada da Ajuda, 1349-017 Lisbon, Portugal; arseniop@isa.ulisboa.pt; 3 Faculty of Pharmacy, Research Institute for Medicines (iMed.ULisboa), Universidade de Lisboa, Av. Prof. Gama Pinto, 1649-003 Lisbon, Portugal; joanalmg@hotmail.com (J.G.); cmprodrigues@ff.ulisboa.pt (C.M.P.R.)

**Keywords:** invasive species, *Acacia melanoxylon*, terpenoids, HCT116 human colorectal cancer cells, cytotoxicity

## Abstract

Invasive species are currently a world menace to the environment, although the study of their chemistry may provide a means for their future beneficial use. From a study of Portuguese *Acacia melanoxylon* R. Br. five known compounds were isolated: lupeol, 3β-*Z*-coumaroyl lupeol, 3β-*E*-coumaroyl lupeol (dioslupecin A), kolavic acid 15-methyl ester and vomifoliol (blumenol A). Their structures were elucidated by 1D and 2D NMR spectroscopy and mass spectrometry, and as a result some corrections are made to their previous ^13^C NMR assignments. Cytotoxicity of 3β-*E*-coumaroyl lupeol (dioslupecin A) and kolavic acid 15-methyl ester was evaluated against HCT116 human colorectal cancer cells although biological activity was not evident.

## 1. Introduction

Plant invasive species are one of the great threats to biodiversity since they establish and supersede native species, leading occasionally to the extinction of the latter, by disrupting the biotic and abiotic balance of the invaded ecosystem. Apart from this ecological impact, they also have a socio-economic impact by influencing human health, infrastructures and local economies [1,2]. These species have long been a concern in the Portuguese territory [3,4,5] and elsewhere, which resulted in the publication of legal regulations to prevent and manage the introduction and spread of invasive alien plants, both at the national [6] and European level [7,8]. The management of this problem in Europe costs millions of euros [9] and, since eradication is rarely achieved, actions end up frequently with periodic growth control and containment of these species [10]. The use of invasive species as a source of chemicals or pharmaceuticals allows a rational use of resources that can mitigate the cost of their control, turning a useless and abundant natural good into an added value resource [11]. Most likely, the prevalence of invasive species over endemic ones relies on the bioactivity of the metabolites they produce as invaders, that are surely responsible for their ease of expansion and dominance of the new habitat [11,12].

Bearing this in mind, we began our studies on the chemistry of invasive *Acacia* species. *Acacia* species, mainly from Australia, settled in the Mediterranean area and conquered significant lands. In Portugal they are distributed throughout the country, preferentially on acid substrates. Originally, they were introduced as a source for wood, as an erosion preventer, for reforestation purposes and for ornamental reasons and the perfume industry, among others [13,14]. Abiotic and biotic factors favored their establishment and with time some of their uses deteriorated and economic value lowered, leading to an increase in their abundance and to an invasion condition [14,15,16]. The management and control of Acacia invasions includes various steps, from risk assessment to containment, eradication and ecosystem restoration actions [13,15,17]. Some of the containment and control actions include mechanical removal (ring-barking, hand-pulling or cutting) followed by chemical control (with glyphosate), or biological control [18].

*A. melanoxylon* R. Br. (Australian blackwood) is a 15 m tree with evergreen leaves and pale-yellow flowers arranged in a globular head of 10–12 mm diameter. Flowering occurs in Portugal from February to June, and its fruits are brownish red pods. The seeds, encircled by an orange funicle, remain viable in the ground for more than 50 years, are dispersed by birds, wind, water, or rodents, and germinate after a space opening and/or fire occurrence. This species also propagates vegetatively, forming vigorous sprouts from the stump and roots [19].

Previous studies on the chemistry of *A. melanoxylon* include the isolation of hyperoside (quercetin-3-d-galactoside) from the flowers [20], dihydroflavonoids [21], hydroxyflavans [22], leucoanthocyanidins [23,24,25], a pyrogallol A-ring proanthocyanidin dimer [26], [4-*O*-4]-linked biflavanoids [27] from the heartwood, and acamelin (a furanoquinone) and a benzoquinone from undisclosed parts [28]. Studies from Portuguese invasive species include the identification of Δ^7^ phytosterols, phytosteryl glucosides and long-chain *n*-alkyl caffeates by GC-EIMS from the dichloromethane extracts of wood and bark [29,30], as well as the antimicrobial activity of aqueous, ethanolic and methanolic leaf extracts, that showed no interesting results [31]. Other references to bioactivity of extracts of this species can be found in the recent review by Correia et al. on the biomass valorization of *Acacia* species [32].

Following our interest in the chemistry of invasive species, in this study we report on the isolation of five compounds: lupeol **1** [33], 3β-*Z*-coumaroyl lupeol **2** [34,35], 3β-*E*-coumaroyl lupeol (dioslupecin A) **3** [36,37], kolavic acid 15-methyl ester **4** [38,39,40] and vomifoliol (blumenol A) **5** [41,42,43] from a Portuguese invasive *A. melanoxylon* and demonstrate the utility of investigating its phytoconstituents, and as corollary of invasive species in general.

For compound **3**, previous biological activity studies regard the cytotoxicity studies on KB, COLO-205, HEPA-3B, and HELA cell lines and showed no activity [37].

For compound **4**, biological activity studies have been performed, namely inhibitory (*Trypanosoma brucei*) [38], and antimicrobial (*Escherichia coli*, *Proteus* sp., *Streptococcus aureus* and *Candida albicans*) [39] activities, as well as cytotoxicity (AGP01, HCT116, MCF07, NIHOVCAR, SKAMELL4 and SF295 cell lines) and anti-inflammatory activities [40]. Although antimicrobial activities and cytotoxicity were not observed, compound **4** showed high lipoxygenase inhibition activity when compared to standard quercertin and inhibited the production of IL-6 [40]. It also exhibited an inhibitory activity on the growth of *Trypanosoma brucei* with respect to the clinically used antitrypanosomal agents suramin and melarsoprol and showed a strong and selective inhibitory activity on the GAPDH enzyme of *T. brucei* [38].

For compound **5**, previous anticancer studies showed no significant activity (HIF-1 and NF-κB activities in reporter assays, and in cytotoxicity against A549, MDA-MB-231, MCF-7, KB, KB-VIN, HT29, A498, PC3 and PACA2 cell lines) [43,44,45]. Antimicrobial (*Micrococcus tetragenus*, *Escherichia coli*, *Staphylococcus albus*, *Bacillus cereus*, *Staphylococcus aureus*, *Micrococcus luteus*, *Bacillus subtilis*, *Pseudomonas aeruginosa*, methicillin-resistant *Staphylococcus aureus*, *Vibrio parahemolyticus* and *Candida albicans*), DPPH free radical scavenging, acetylcholinesterase inhibitory and brine shrimp larvicidal activities also showed no results [42,46].

## 2. Results and Discussion

### 2.1. Compound Identification

**Figure 1:**
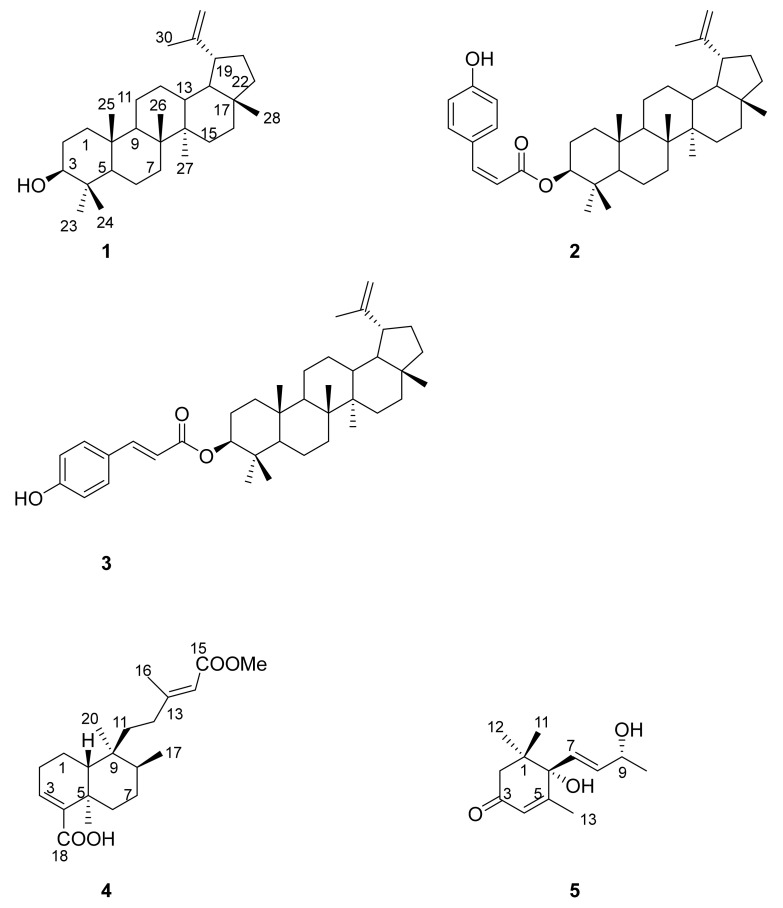
Lupeol **1**, 3β-*Z*-coumaroyl lupeol **2**, 3β-*E*-coumaroyl lupeol (dioslupecin A) **3**, kolavic acid 15-methyl ester **4** and vomifoliol (blumenol A) **5** isolated from *A. melanoxylon*.

From the dichloromethane extract (17.63 g) of *A. melanoxylon* R. Br. (1250 g) collected at Peninha, Sintra, Portugal, lupeol **1** [33] (purified, <0.1 mg), 3β-*Z*-coumaroyl lupeol **2** [34,35] (purified, 15.6 mg), 3β-*E*-coumaroyl lupeol (dioslupecin A) **3** [36,37] (purified, 39.0 mg), and kolavic acid 15-methyl ester **4** [38,39,40] (purified 111.1 mg) were isolated (Figure 1). The study of the alkaloid content of this species led instead to the isolation of vomifoliol (blumenol A) **5** [41,42,43] (unpurified, 5.4 mg). Alkaloids were detected by TLC but their isolation was not achieved due to the low content in this species (this may be related to the time of harvest, since the accumulation of alkaloids is known to be seasonal, or due to other biotic or abiotic factors).

The use of 1D and 2D NMR allowed the structure determination of all five compounds, further confirmed by mass spectrometry and comparison with literature data (Figure 1).

Compound **1** was identified by its characteristic H-3 (δ 3.19 ppm, *dd*, *J* = 5.0 Hz, *J* = 11.2 Hz), aliphatic methyl groups (δ 0.76 ppm, 0.79 ppm, 0.83 ppm, 0.94 ppm, 0.97 ppm and 1.03 ppm), CH_2_-29 (δ 4.56 ppm, *sl* and δ 4.56 ppm, *sl*) and Me-30 (δ 1.68 ppm) resonances (Figure S1), all in agreement with literature values [33]. Comparison of the NMR spectra of **2** (Figures S2–S8) and **3** (Figures S9–S14) with that of **1** (Figure S1) allowed the recognition of a lupeol base skeleton substituted at C-3 with a coumaroyl unit—the structures of 3β-*Z*-coumaroyl lupeol **2** [34,35] and 3β-*E*-coumaroyl lupeol (dioslupecin A) **3** [36,37] were proposed, based on the *J* value couplings of the substituent’s double bond (12.7 Hz and 15.9 Hz, respectively). Further confirmation came from comparison of the ^1^H and ^13^C resonance values with those of the literature [34,35,36,37].

For compound **3** corrections are made for the literature [36,37] resonances of the aliphatic methyl groups, based on HMBC and NOESY correlations (Table 1): HMBC between δ 0.89 and δ 0.92 with δ 81.2 (C-3) clearly indicates the presence of Me-23 and Me-24, distinguished by NOESY with H-3 (for δ 0.89); HMBC of δ 0.88 with δ 50.3 (C-9) and δ 37.1 (C-10) assigns Me-25; HMBC of δ 1.03 with δ 34.2 (C-7), δ 40.8 (C-8), δ 42.8 (C-14) and δ 50.3 (C-9) assigns Me-26; HMBC of δ 0.95 with δ 27.4 (C-15) and δ 42.8 (C-14) assigns Me-27; finally, HMBC of δ 0.79 with δ 35.5 (C-16), δ 43.0 (C-17) and δ 48.2 (C-18) assigns Me-28.

On purification and analysis, isomerization of the double bond was observed as described previously for coumaroyl esters [47]: the final ^1^H NMR spectrum of the sample of **2** is composed of a 2.0:1.0 mixture of *E* and *Z* isomers (Figure S15), also present in the chromatogram of the GC-FID analysis (1.6:1.0, different ratios accountable by different isomerization times, Figure S16). Even the *E* isomer, compound **3**, obtained in pure form, seems to equilibrate in CDCl_3_ to a 4.4:1.0 mixture of *E* and *Z* forms (Figure S17).

For compound **4**, a detailed analysis of the NMR data led to the proposed structure, confirmed by the [M − H_2_O + H]^+^ and [M − H]^−^ ions present at *m/z* 331.3 u and *m/z* 347.2 u in the positive and negative ESI-MS spectra, respectively (Figures S18–S25). Analysis of the spectra and comparison of the NMR resonances with those of the literature [38,39,40] allowed us to make some corrections to previously assigned ^13^C NMR values that should be interchanged: (a) C-2 and C-7: interchanged based on HMBC with H-3 and Me-17, respectively; (b) C-6 and C-11: interchanged based on COSY of H-12 with H-11 (C-11, assigned by HSQC); (c) Me-19 and Me-20: interchanged based on HMBC with C-11; (d) C-5 and C-9: interchanged based on HMBC correlations of Me-19 and Me-20, respectively; and (e) C-15 and C-18: interchanged based on HMBC of the OMe group (confirmed by HMBC of H-14). Table 2 lists the full ^13^C NMR assignment and the ^1^H NMR outstanding resonances (full ^1^H NMR assignment can be found in the literature [38,39,40]).

Finally, for compound **5**, the proposed structure based on NMR analysis was confirmed by the [M − H_2_O + H]^+^, [M + H]^+^, [M + Na]^+^ and [M − H]^−^ ions present at *m/z* 207.1 u, *m/z* 225.1 u, *m/z* 247.1 u, and *m/z* 223.0 u in the positive and negative ESI-MS spectra, respectively (Figures S26–S32). Comparison of the ^1^H and ^13^C NMR resonances with those of the literature [41,42,43] confirm the structure, and allows us to correct the ^13^C NMR δ values of the Δ^7^ double bond that must be interchanged: C-7 δ 129.0 ppm and C-8 δ 135.7 ppm (ascertained by HSQC).

### 2.2. Cytotoxicity Evaluation

Cytotoxicity studies were performed for kolavic acid 15-methyl ester **4** and a sample of 3β-*E*-coumaroyl lupeol (dioslupecin A) **3**. The fact that compounds **2** and **3** equilibrate when in solution prevented us from testing both compounds separately. Since lupeol **1** and vomifoliol (blumenol A) **5** were isolated in small amounts their biological testing was also not performed.

To determine cellular toxicity, preliminary assays were performed with compounds **3** and **4** incubated in HCT116 cells for 72 h. Compound **3** showed to decrease cell viability to 64% only at the highest concentration tested (243 µM). However, at the same concentration, compound **4** decreased cell viability to 3%. 

**Figure 2:**
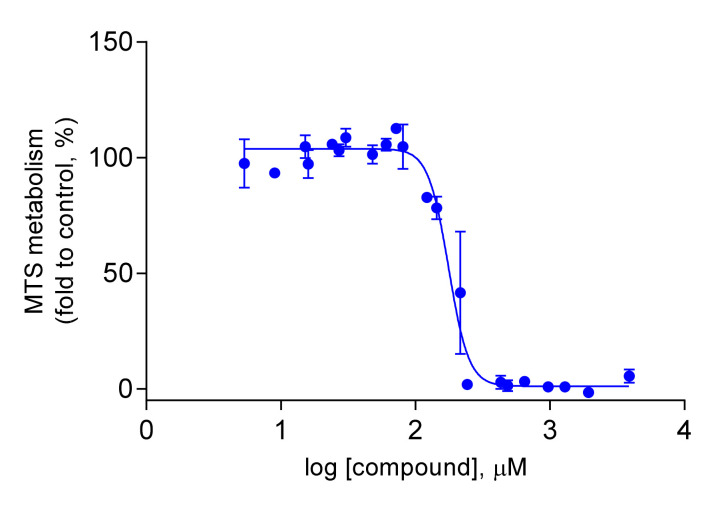
Dose-response curve in HCT116 cells upon incubation with compound **4** for 72 h. These results are representative of at least three independent experiments.

**Figure 3:**
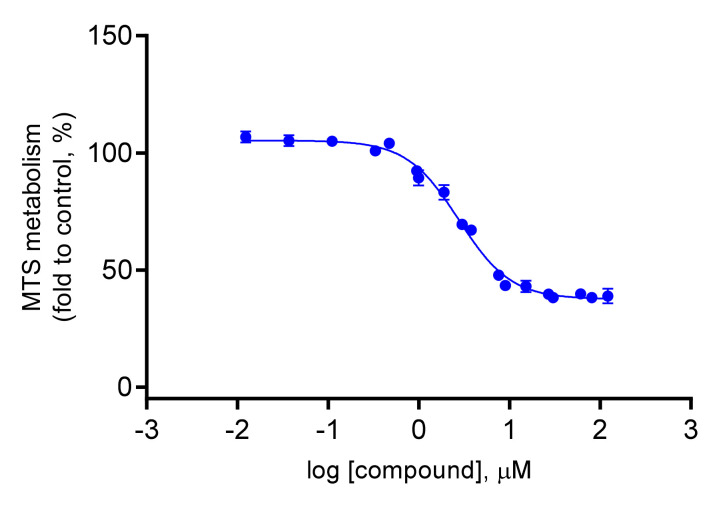
Dose-response curve in HCT116 cells upon incubation with 5-FU for 72 h. These results are representative of at least three independent experiments.

Therefore, only the IC50 of compound **4** was determined by the MTS metabolism assay, in order to evaluate its potential cytotoxic activity. In fact, compound **4** showed to have a relatively low cytotoxicity against the HCT116 colon cancer cell line, with an IC50 value of 176.3 µM (95% CI = 163.8 to 189.8 µM, Figure 2). This might suggest its use as an anti-inflammatory agent [40]. Nonetheless, additional studies using other cell lines are required to discard completely the cytotoxic effect of this compound. 5-Fluorouracil (5-FU), a cytotoxic agent in colon cancer treatment, was used as a positive control (IC50 value of 2.763 µM; 95% CI = 2.539 to 3.007 µM, Figure 3).

## 3. Materials and Methods

### 3.1. General Experimental Procedures

Silica gel 60 for chromatography (Merck 7734 or Merck 109385) was used for column chromatography (gravity and flash, respectively) and TLC was performed on Silica gel 60 Merck 5744 (0.5 mm) or 5554 TLC plates employing 254 nm and/or 366 nm UV-lamp for visualization. Molybdophosphoric acid or Munier Dragendorff reagent were used for additional spot development [48]. NMR spectra were recorded on a 400 MHz (100 MHz for ^13^C) Brucker Avance III 400 or 500 MHz (125 MHz for ^13^C) Bruker AVANCE Neo 500 spectrometer (Fällanden, Switzerland) at 25 °C using standard pulse programs. Residual solvent signals were used for calibration (^1^H: δ(CHCl_3_) = 7.26 ppm, ^13^C: δ(CDCl_3_) = 77.00 ppm). GC-FID was performed on GC Agilent 6890 operated at the following conditions: carrier gas Helium, split ratio 1:30; Column: ZB1HTinferno, L: 15 m, φ: 0.25 mm, df: 0.10 μm, injector temperature 280 °C; detector temperature 280 °C; temperature program 100 °C, hold for 0 min, increase 10 °C/min to 320 °C, hold for 20 min. 

ESI-MS spectra were performed on a Thermo orbitrap Qexactive focus apparatus with direct inlet by a Thermo vanquish apparatus.

### 3.2. Plant Collection and Preparation

Branches and leaves of *A. melanoxylon* R. Br. were collected at Peninha, Sintra, Portugal (38°46′10″ N 9°27′33″ W), on 15 December 2019 and a voucher specimen (LISI032915, Patrícia Máximo and Ana Lourenço, *s.n*., collected on 1 March 2020) was deposited at Herbário João de Carvalho e Vasconcellos (LISI), School of Agriculture (ISA), University of Lisbon. After air drying at room temperature in the dark, and milling, 1850 g were obtained.

### 3.3. Extraction and Isolation

#### 3.3.1. Dichloromethane Extract

An amount of 1250 g of *A. melanoxylon* R. Br. was defatted with *n*-hexane and extracted with 2.5 L of dichloromethane (DCM), at room temperature for 5 h 30 min. After this time the plant material was separated and re-extracted with another 2.5 L of dichloromethane, at room temperature, for 18 h. The gathered filtrates were evaporated to yield 17.63 g of extract.

The extract was chromatographed on gravity column (diameter 15.0 cm, height 25 cm) with mixtures of *n*-hexane/ethyl acetate, ethyl acetate, two mixtures of ethyl acetate/methanol and methanol to yield 7 fractions (DCM1 (8/2), DCM2 (7/3), DCM3 (6/4), DCM4 (1/1), DCM5 (EtOAc), DCM6 (10% and 20% of MeOH), DCM7 (MeOH)). 

The fraction DCM1 (4.13 g) was purified by flash chromatography with a mixture of *n*-hexane/ethyl acetate 9/1 to yield six fractions, two of them further purified: DCM1E and DCM1F. The fraction DCM1E (638.0 mg) was purified by flash column chromatography with mixtures of *n*-hexane/ethyl acetate 85/15 to yield the fraction DCM1E1 (586.0 mg). This fraction was further purified by flash column chromatography with mixtures of n-hexane/ethyl acetate 85/15, 8/2, and ethyl acetate to yield 4 fractions. One of them, DCM1E1B (385.0 mg), was purified twice by thin layer chromatography using mixtures of n-hexane/ethyl acetate 8/2 and 85/15 to yield fraction DCM1E1Bp1p, compound **1** (15.1 mg). Another, DCM1E1C (30.0 mg) was purified by thin layer chromatography using a mixture of *n*-hexane/ethyl acetate 8/2 to yield DCM1E1Cp1, compound **2** (23.1 mg). Another, DCM1E1D (64.0 mg) gave a mixture of compounds **2** and **3**. The fraction DCM1F (574.0 mg) was purified by flash column chromatography with a mixture of *n*-hexane/ethyl acetate 85/15 to yield fraction DCM1F2 (393.0 mg) that, after purification by thin layer chromatography using a mixture of *n*-hexane/ethyl acetate 8/2, yielded fraction DCM1F2p, compound **3** (57.0 mg).

The fraction DCM2 (2.91 g) was purified by flash column chromatography with mixtures of *n*-hexane/ethyl acetate 8/2 and 7/3 to yield three fractions; one of them, DCM2B (1.00 g), was further purified by flash column chromatography using mixtures of *n*-hexane/ethyl acetate 8/2 and 7/3, and ethyl acetate to yield six fractions; one of them, DCM2B5 yielded compound **4** (168.8 mg).

Final purification of the compounds for biological testing was achieved by flash chromatography for **4** (white solid, 111.1 mg, *n*-hexane/ethyl acetate 8/2) or thin layer chromatography for **1** (white solid, <1 mg, *n*-hexane/ethyl acetate 8/2), **2** (white solid, 3.5 mg, *n*-hexane/ethyl acetate 8/2), **3** (white solid, 27.8 mg, *n*-hexane/ethyl acetate 7/3), and for the mixture of **2** and **3** (white solids, 12.1 and 11.2 mg, respectively, *n*-hexane/ethyl acetate 8/2). On performing spectroscopic analysis isomerization of **2** and **3** was observed for some of the samples. 

#### 3.3.2. Vomifoliol 5 Extraction

Acidic extraction of the plant was performed for the isolation of alkaloids. However, a co-extraction compound, vomifoliol (blumenol A) **5**, was isolated instead. An amount of 500 g of *A. melanoxylon* R. Br. was extracted with 1.55 l of HCl 0.5 M, at room temperature, for 40 min. After centrifugation the supernatant was concentrated and basified with NH_4_OH 1M. This solution was applied in an isolute^®^HM-N (Biotage, Uppsala, Sweden) column and after elution with dichloromethane and evaporation, the extract Amx (80.4 mg) was obtained.

This extract was purified by thin layer chromatography using a mixture of dichloromethane/methanol/NH_4_OH 95/5/1 to yield 3 fractions. Of these, Amx-2 (11.8 mg) was purified by thin layer chromatography using a mixture of dichloromethane/methanol/NH_4_OH 95/5/1 to yield fraction Amx-2.2B, compound **5** (5.4 mg).

### 3.4. Compound Characterization

Compound **1** (unpurified)—^1^H NMR spectrum, see Supplementary Materials, Figure S1. Compound **2** (unpurified)—NMR spectra, see Supplementary Materials Figures S2–S8. Compound **3** (unpurified)—NMR spectra, see Supplementary Materials Figures S9–S14. Mixture 1.0:2.0 of compounds **2** and **3**—^1^H NMR spectrum, see Supplementary Materials Figure S15. Mixture 1.0:1.6 of compounds **2** and **3**—GC-FID chromatogram, see Supplementary Materials Figure S16. Mixture 1.0:4.4 of compounds **2** and **3**—^1^H NMR spectrum, see Supplementary Materials Figure S17. Compound **4**—NMR spectra and ESI-MS spectra, see Supplementary Materials Figures S18–S25. Compound **5** (unpurified)—NMR spectra and ESI-MS spectra, see Supplementary Materials Figures S26–S32.

### 3.5. Cell Culture and Treatments

HCT116 human colon carcinoma cells, commonly used in drug screens, were grown in McCoy’s 5A modified medium supplemented with 10% heat-inactivated fetal bovine serum (FBS) and 1% antibiotic/antimycotic solution (Gibco, Life Technologies, Paisley, UK). Cells were cultured at 37 °C under a humidified atmosphere of 5% CO*2*.

For cell viability experiments, HCT116 cells were seeded in 96-well plates, at a concentration of 5 × 10^3^ cells/well and allowed to adhere for 24 h. Then, cells were exposed to the test compound, compound **4**, previously prepared in sterile DMSO. In order to plot a dose–response curve, cells were exposed to this compound in a range of concentrations between 0.04 μM and 4000 μM for 72 h. 5-Fluorouracil (5-FU), a cytotoxic agent used in colon cancer treatment, was used as a positive control and DMSO was used as vehicle control. Data are representative of three independent experiments.

### 3.6. Viability Assays

Cell viability of cells treated with compound **4** was evaluated using the CellTiter 96 AQueous Non-Radioactive Cell Proliferation Assay (Promega, Madison, WI, USA) according to the manufacturer’s instructions. This colorimetric assay is based in the capacity of metabolic active cells to convert 3-(4,5-dimethylthiazo-2-yl)-5-(3-carboxymethoxyphenyl)-2-(4-sulfophenyl)-2H-tetrazolium inner salt (MTS) to formazan, a dye that is soluble in cell culture media. Formazan is quantified by measuring the absorbance at 490 nm and correlates with the amount of living cells in culture. Absorbance was measured using the GloMax-Multi+ microplate multimode reader (Promega, Madison, WI, USA) and the best-fit IC50 value, from at least three independent experiments, was calculated using the log (inhibitor) versus response (variable slope) function from GraphPad Prism software (version 8.0.2; San Diego, CA, USA).

## 4. Conclusions

In this study, five already known compounds were isolated and as a result some corrections are made to their previous ^13^C NMR assignments. Cytotoxicity against HCT116 cells was evaluated for two of them, although no positive results were obtained.

As for the utility of the study of the chemistry of invasive species, here illustrated by two extracts of *A. melanoxylon*, we can propose that they can be used as a source of bioactive metabolites. Based on the literature and our own experimental results, lupeol derivatives **2** and **3** show no anticancer activity. Previous reports on vomifoliol (blumenol A) **5** for diverse bioactivities also showed no results. Nonetheless, kolavic acid 15-methyl ester **4**, the most abundant metabolite, is not cytotoxic and has previously been recognized as a bioactive naturally occurring trypanocide that may contribute to anti-inflammatory effects. *A. melanoxylon* can thus be considered a source for this metabolite.

We further add that it would be interesting to perform a detailed phytochemical study of bark samples of this species in general—ring-barking is presently one of the control measures for this species. Although Freire et al. [29,30] showed the presence of Δ^7^ phytosterols and phytosteryl glucosides in the dichloromethane extracts of the bark by GC-EIMS, some of them with interesting reported bioactivities, many compounds may have escaped this screening; furthermore, although the antimicrobial bioactivity of the more polar ethanolic and aqueous extracts of the bark of this species is not noteworthy [49], again a detailed phytochemical approach could provide pure metabolites for which many more activities could be considered.

To conclude, although more studies are needed, this paper demonstrates that studying the chemistry of invasive species might provide a utility for this natural and abundant good that is currently underexplored.

## Figures and Tables

**Table 1:** ^1^H and ^13^C resonances of the aliphatic methyl groups of 3β-*E*-coumaroyl lupeol **3** (CDCl_3_, 400 and 100 MHz).

	^1^H (δ ppm)	^13^C (δ ppm) *
Me-23	0.89	28.0
Me-24	0.92	16.6
Me-25	0.88	16.2
Me-26	1.03	15.9
Me-27	0.95	14.5
Me-28	0.79	18.0

* assigned by HSQC.

**Table 2:** ^13^C assignment and the ^1^H outstanding resonances of kolavic acid 15-methyl ester **4** (CDCl_3_, 125 and 500 MHz). *s* singlet, *d* duplet, *t* triplet.

	^1^H	^13^C		^1^H	^13^C
1		16.8	11		36.2
2		24.4	12		34.4
3	6.80 *t* 3.8	142.2	13		161.5
4		137.5	14	5.68 *d* 1.0	114.9
5		36.3	15		167.3
6		36.8	16	2.18 *d* 1.2	19.2
7		28.6	17	0.77 *d* 6.9	15.9
8		37.8	18		172.7
9		40.3	19	1.24 *s*	33.4
10		45.4	20	0.78 *s*	18.0
			COOMe	3.69 *s*	50.8

## Data Availability

Not applicable.

## Supplementary Materials

The following are available online at https://www.mdpi.com/article/10.3390/plants10122698/s1. Figure S1—^1^H NMR spectrum of Lupeol **1** (unpurified) (CDCl_3_, 400 MHz); Figure S2—^1^H NMR spectrum of 3β-*Z*-coumaroyl lupeol **2** (unpurified) (CDCl_3_, 400 MHz); Figure S3—^13^C NMR spectrum of 3β-*Z*-coumaroyl lupeol **2** (unpurified) (CDCl_3_, 100 MHz); Figure S4—DEPT135 spectrum of 3β-*Z*-coumaroyl lupeol **2** (unpurified) (CDCl_3_); Figure S5—HSQC spectrum of 3β-*Z*-coumaroyl lupeol **2** (unpurified) (CDCl_3_); Figure S6—HMBC spectrum of 3β-*Z*-coumaroyl lupeol **2** (unpurified) (CDCl_3_); Figure S7—COSY spectrum of 3β-*Z*-coumaroyl lupeol **2** (unpurified) (CDCl_3_); Figure S8—NOESY spectrum of 3β-*Z*-coumaroyl lupeol **2** (unpurified) (CDCl_3_); Figure S9—^1^H NMR spectrum of 3β-*E*-coumaroyl lupeol (dioslupecin A) **3** (unpurified) (CDCl_3_, 400 MHz); Figure S10—^13^C NMR spectrum of 3β-*E*-coumaroyl lupeol (dioslupecin A) **3** (unpurified) (CDCl_3_, 100 MHz); Figure S11—DEPT135 spectrum of 3β-*E*-coumaroyl lupeol (dioslupecin A) **3** (unpurified) (CDCl_3_); Figure S12—HSQC spectrum of 3β-*E*-coumaroyl lupeol (dioslupecin A) **3** (unpurified) (CDCl_3_); Figure S13—HMBC spectrum of 3β-*E*-coumaroyl lupeol (dioslupecin A) **3** (unpurified) (CDCl_3_); Figure S14—NOESY spectrum of 3β-*E*-coumaroyl lupeol (dioslupecin A) **3** (unpurified) (CDCl_3_); Figure S15—^1^H NMR spectrum of the mixture of 3β-*Z*-coumaroyl lupeol **2** and 3β-*E*-coumaroyl lupeol **3** (1.0:2.0) (CDCl_3_, 500 MHz); Figure S16—GC-FID chromatogram of the mixture of 3β-*Z*-coumaroyl lupeol **2** and 3β-*E*-coumaroyl lupeol **3** (1.0:1.6); Figure S17—^1^H NMR spectrum of the mixture of 3β-*Z*-coumaroyl lupeol **2** and 3β-*E*-coumaroyl lupeol **3** (1.0:4.4) (CDCl_3_, 500 MHz); Figure S18—^1^H NMR spectrum of kolavic acid 15-methyl ester **4** (CDCl_3_, 500 MHz); Figure S19—^13^C NMR spectrum of kolavic acid 15-methyl ester **4** (CDCl_3_, 125 MHz); Figure S20—DEPT135 spectrum of kolavic acid 15-methyl ester **4** (CDCl_3_); Figure S21—HSQC spectrum of kolavic acid 15-methyl ester **4** (CDCl_3_); Figure S22—HMBC spectrum of kolavic acid 15-methyl ester **4** (CDCl_3_); Figure S23—COSY spectrum of kolavic acid 15-methyl ester **4** (CDCl_3_); Figure S24—NOESY spectrum of kolavic acid 15-methyl ester **4** (CDCl_3_); Figure S25—ESI-MS spectra of kolavic acid 15-methyl ester **4**; Figure S26—^1^H NMR spectrum of vomifoliol (blumenol A) **5** (unpurified) (CDCl_3_, 500 MHz); Figure S27—^13^C NMR spectrum of vomifoliol (blumenol A) **5** (unpurified) (CDCl_3_, 125 MHz); Figure S28—DEPT135 spectrum of vomifoliol (blumenol A) **5** (unpurified) (CDCl_3_); Figure S29—HSQC spectrum of vomifoliol (blumenol A) **5** (unpurified) (CDCl_3_); Figure S30—HMBC spectrum of vomifoliol (blumenol A) **5** (unpurified) (CDCl_3_); Figure S31—COSY spectrum of vomifoliol (blumenol A) **5** (unpurified) (CDCl_3_); Figure S32—ESI-MS spectra of vomifoliol (blumenol A) **5** (unpurified).
