# Supplementary material for: Exploring the Phytochemicals of Acacia melanoxylon R. Br
Source: Plants (Basel). 2021 Dec 8;10(12):2698. doi: 10.3390/plants10122698 (PMC8706220; doi:10.3390/plants10122698)

Exploring the Phytochemicals of *Acacia melanoxylon* R. Br.

Diana Alves[1], Sidónio Duarte[1], Pedro Arsénio[2], Joana Gonçalves[3], Cecília M. P. Rodrigues[3], Ana Lourenço[1,]* and Patrícia Máximo[1,]*

Figure S1 - ^{1}H NMR spectrum of Lupeol **1** (unpurified) ($CDCl_3$, 400 MHz) 3

Figure S2 - ^{1}H NMR spectrum of 3β-*Z*-coumaroyl lupeol **2** (unpurified) ($CDCl_3$, 400 MHz) 3
Figure S3 - ^{13}C NMR spectrum of 3β-*Z*-coumaroyl lupeol **2** (unpurified) ($CDCl_3$, 100 MHz) 4
Figure S4 - DEPT135 spectrum of 3β-*Z*-coumaroyl lupeol **2** (unpurified) ($CDCl_3$) 4
Figure S5 - HSQC spectrum of 3β-*Z*-coumaroyl lupeol **2** (unpurified) ($CDCl_3$) 5
Figure S6 - HMBC spectrum of 3β-*Z*-coumaroyl lupeol **2** (unpurified) ($CDCl_3$) 5
Figure S7 - COSY spectrum of 3β-*Z*-coumaroyl lupeol **2** (unpurified) ($CDCl_3$) 6
Figure S8 - NOESY spectrum of 3β-*Z*-coumaroyl lupeol **2** (unpurified) ($CDCl_3$) 6

Figure S9 - ^{1}H NMR spectrum of 3β-*E*-coumaroyl lupeol (dioslupecin A) **3** (unpurified) ($CDCl_3$, 400 MHz) 7
Figure S10 - ^{13}C NMR spectrum of 3β-*E*-coumaroyl lupeol (dioslupecin A) **3** (unpurified) ($CDCl_3$, 100 MHz) 7
Figure S11 - DEPT135 spectrum of 3β-*E*-coumaroyl lupeol (dioslupecin A) **3** (unpurified) ($CDCl_3$) 8
Figure S12 - HSQC spectrum of 3β-*E*-coumaroyl lupeol (dioslupecin A) **3** (unpurified) ($CDCl_3$) 8
Figure S13 - HMBC spectrum of 3β-*E*-coumaroyl lupeol (dioslupecin A) **3** (unpurified) ($CDCl_3$) 9
Figure S14 - NOESY spectrum of 3β-*E*-coumaroyl lupeol (dioslupecin A) **3** (unpurified) ($CDCl_3$) 9

Figure S15 - ^{1}H NMR spectrum of the mixture of 3β-*Z*-coumaroyl lupeol **2** and 3β-*E*-coumaroyl lupeol **3** (1.0:2.0) ($CDCl_3$, 500 MHz) 10
Figure S16 - GC-FID chromatogram of the mixture of 3β-*Z*-coumaroyl lupeol **2** and 3β-*E*-coumaroyl lupeol **3** (1.0:1.6) 11
Figure S17 - ^{1}H NMR spectrum of the mixture of 3β-*Z*-coumaroyl lupeol **2** and 3β-*E*-coumaroyl lupeol **3** (1.0:4.4) ($CDCl_3$, 500 MHz) 12

Figure S18 - ^{1}H NMR spectrum of kolavic acid 15-methyl ester **4** ($CDCl_3$, 500 MHz) 12
Figure S19 - ^{13}C NMR spectrum of kolavic acid 15-methyl ester **4** ($CDCl_3$, 125 MHz) 13

Figure S20 - DEPT135 spectrum of kolavic acid 15-methyl ester **4** ($CDCl_3$) 13
Figure S21 - HSQC spectrum of kolavic acid 15-methyl ester **4** ($CDCl_3$) 14
Figure S22 - HMBC spectrum of kolavic acid 15-methyl ester **4** ($CDCl_3$) 14
Figure S23 - COSY spectrum of kolavic acid 15-methyl ester **4** ($CDCl_3$) 15
Figure S24 - NOESY spectrum of kolavic acid 15-methyl ester **4** ($CDCl_3$) 15
Figure S25 - ESI-MS spectra of kolavic acid 15-methyl ester **4** 16

Figure S26 - ^{1}H NMR spectrum of vomifoliol (blumenol A) **5** (unpurified) ($CDCl_3$, 500 MHz) 17
Figure S27 - ^{13}C NMR spectrum of vomifoliol (blumenol A) **5** (unpurified) ($CDCl_3$, 125 MHz) 17
Figure S28 - DEPT135 spectrum of vomifoliol (blumenol A) **5** (unpurified) ($CDCl_3$) 18
Figure S29 - HSQC spectrum of vomifoliol (blumenol A) **5** (unpurified) ($CDCl_3$) 18
Figure S30 - HMBC spectrum of vomifoliol (blumenol A) **5** (unpurified) ($CDCl_3$) 19
Figure S31 - COSY spectrum of vomifoliol (blumenol A) **5** (unpurified) ($CDCl_3$) 19
Figure S32 - ESI-MS spectra of vomifoliol (blumenol A) **5** (unpurified) 20

Figure S1 - ^{1}H NMR spectrum of Lupeol **1** (unpurified) ($CDCl_3$, 400 MHz)

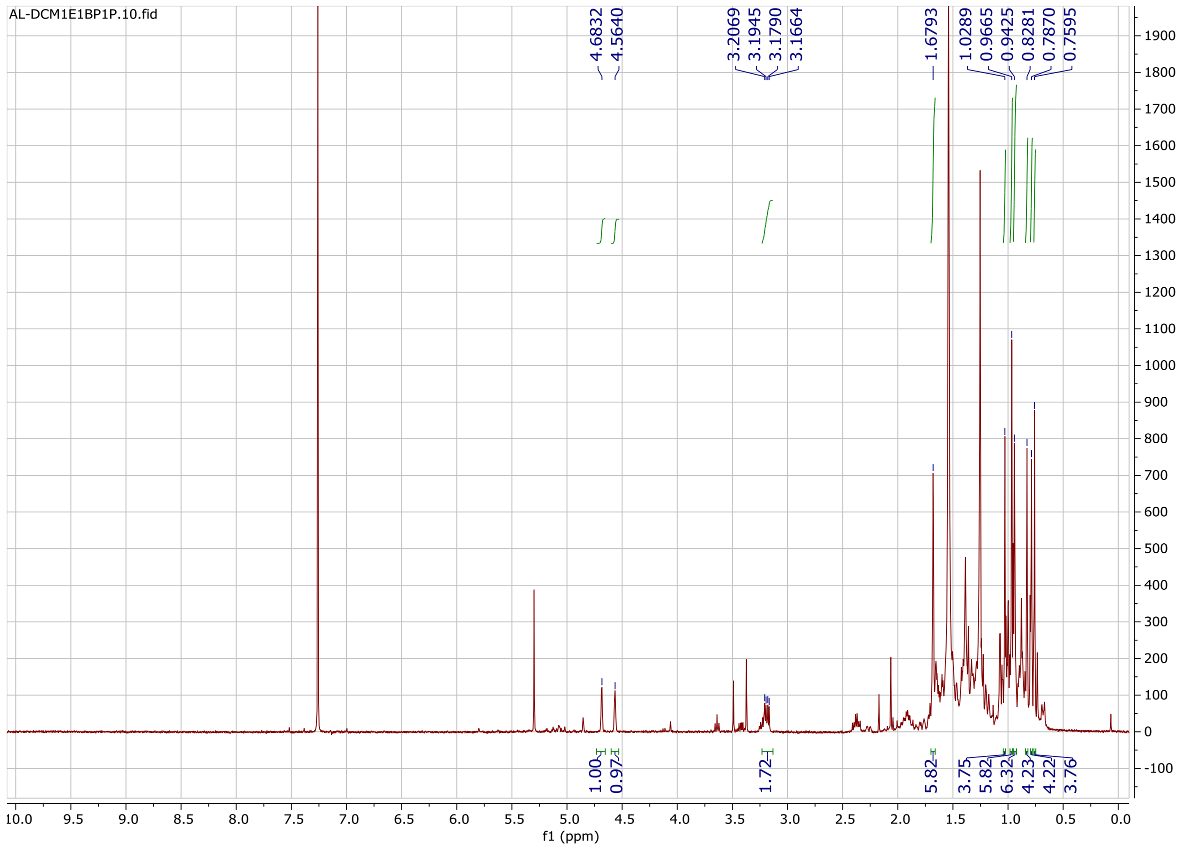

Figure S2 - ^{1}H NMR spectrum of 3β-*Z*-coumaroyl lupeol **2** (unpurified) ($CDCl_3$, 400 MHz)

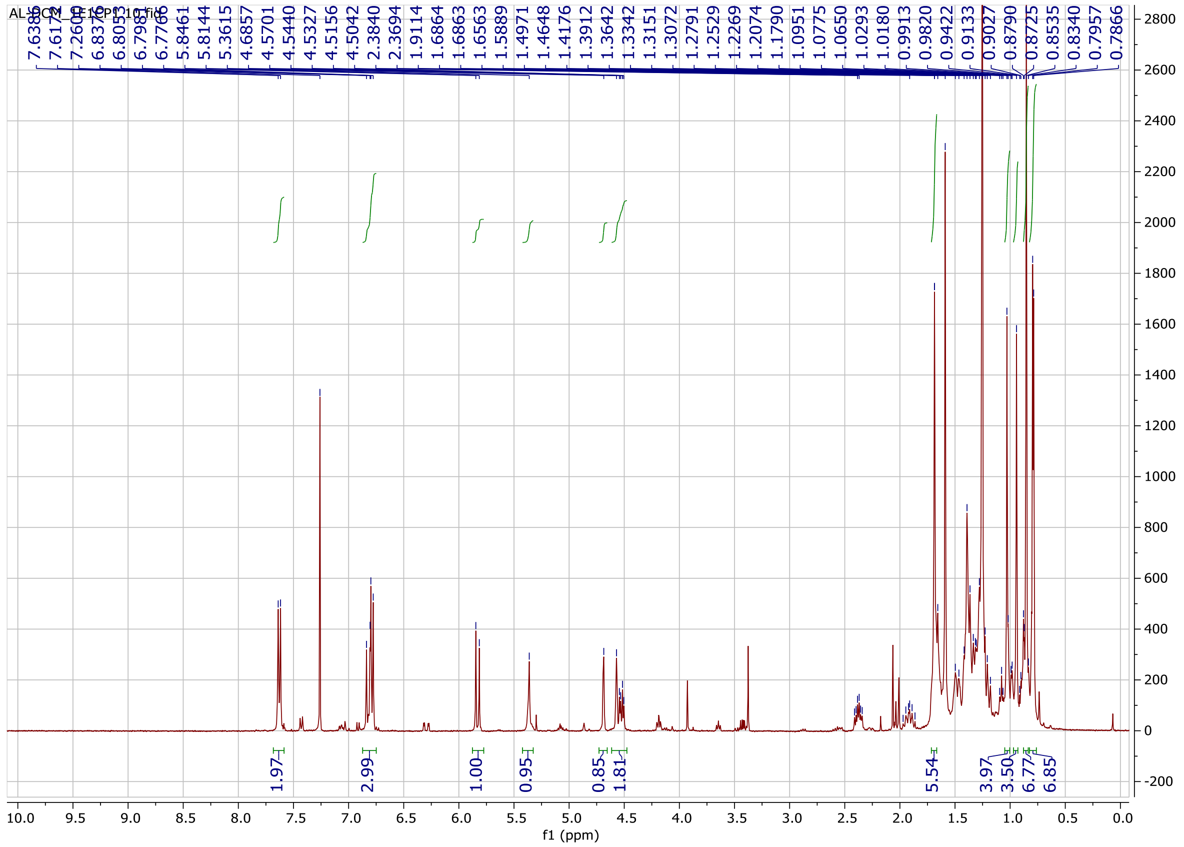

Figure S3 - ^{13}C NMR spectrum of 3β-*Z*-coumaroyl lupeol **2** (unpurified) ($CDCl_3$, 100 MHz)

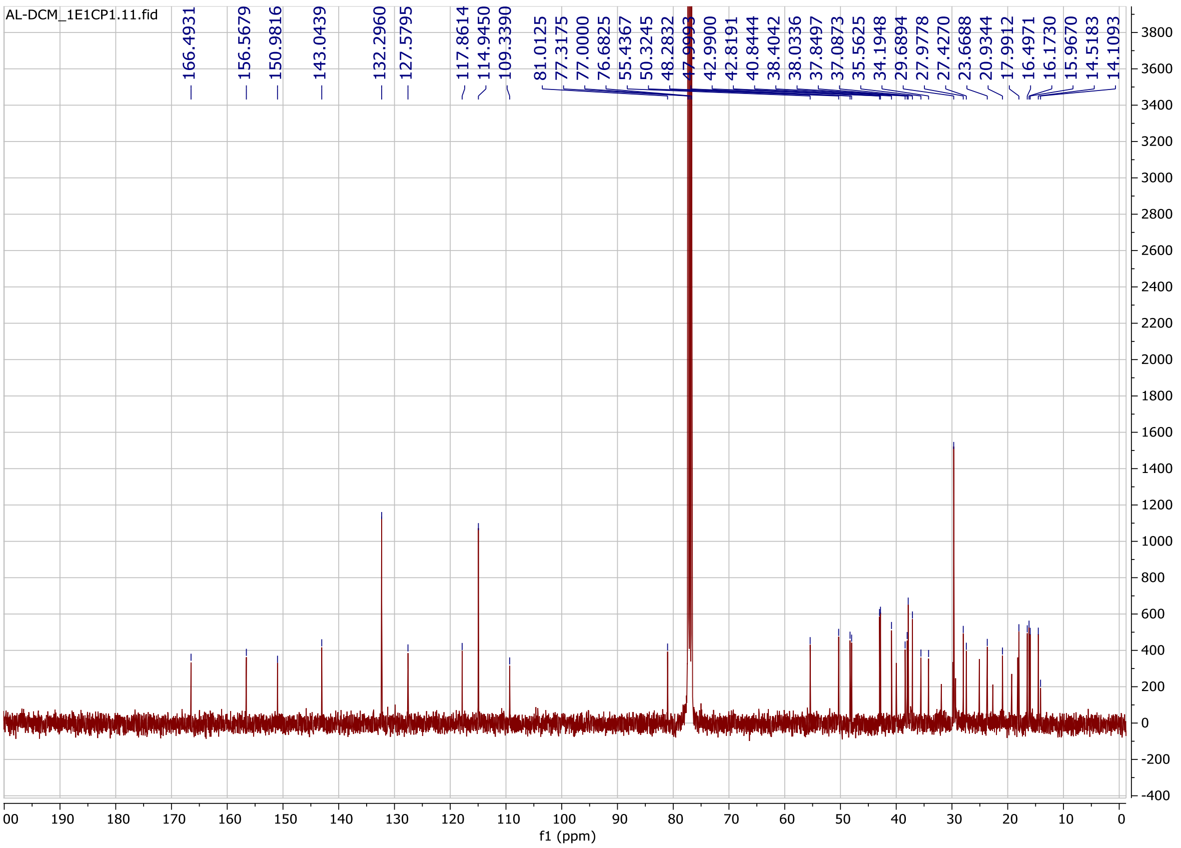

Figure S4 - DEPT135 spectrum of 3β-*Z*-coumaroyl lupeol **2** (unpurified) ($CDCl_3$)

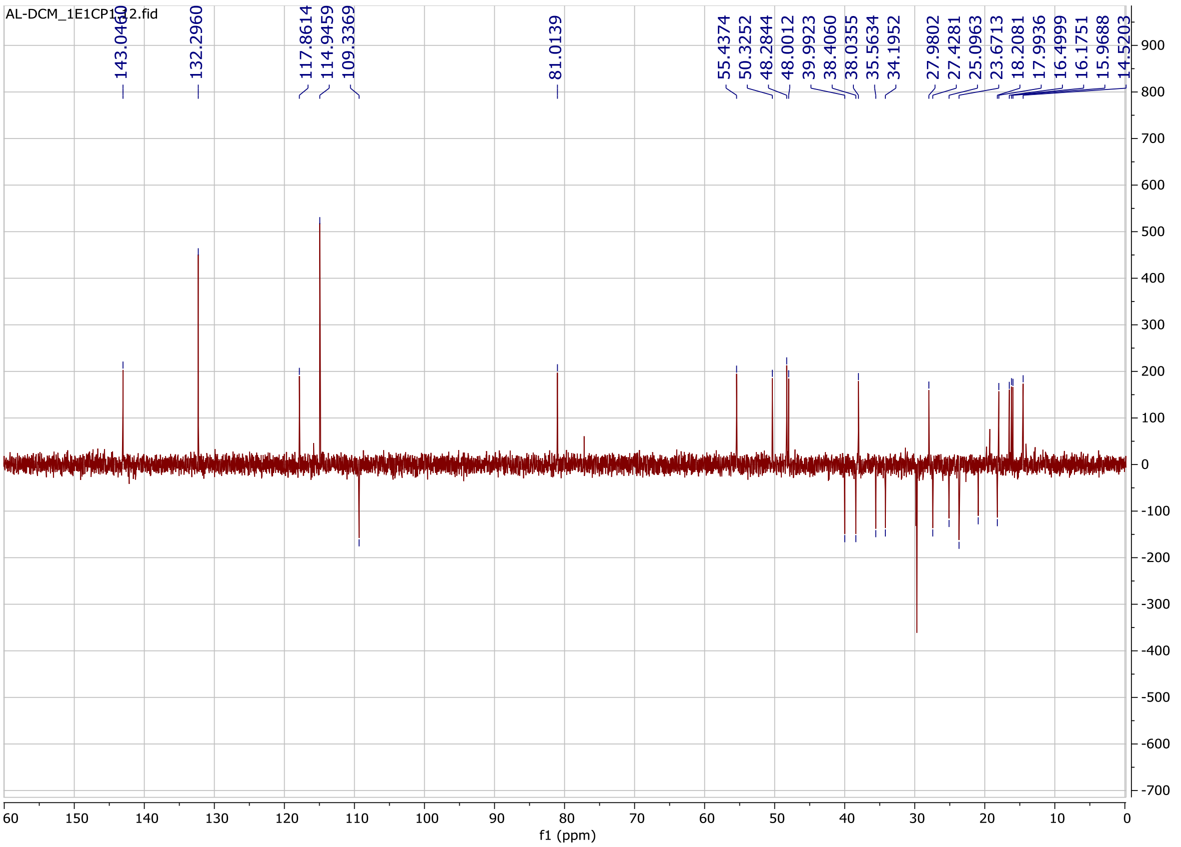

Figure S5 - HSQC spectrum of 3β-*Z*-coumaroyl lupeol **2** (unpurified) ($CDCl_3$)

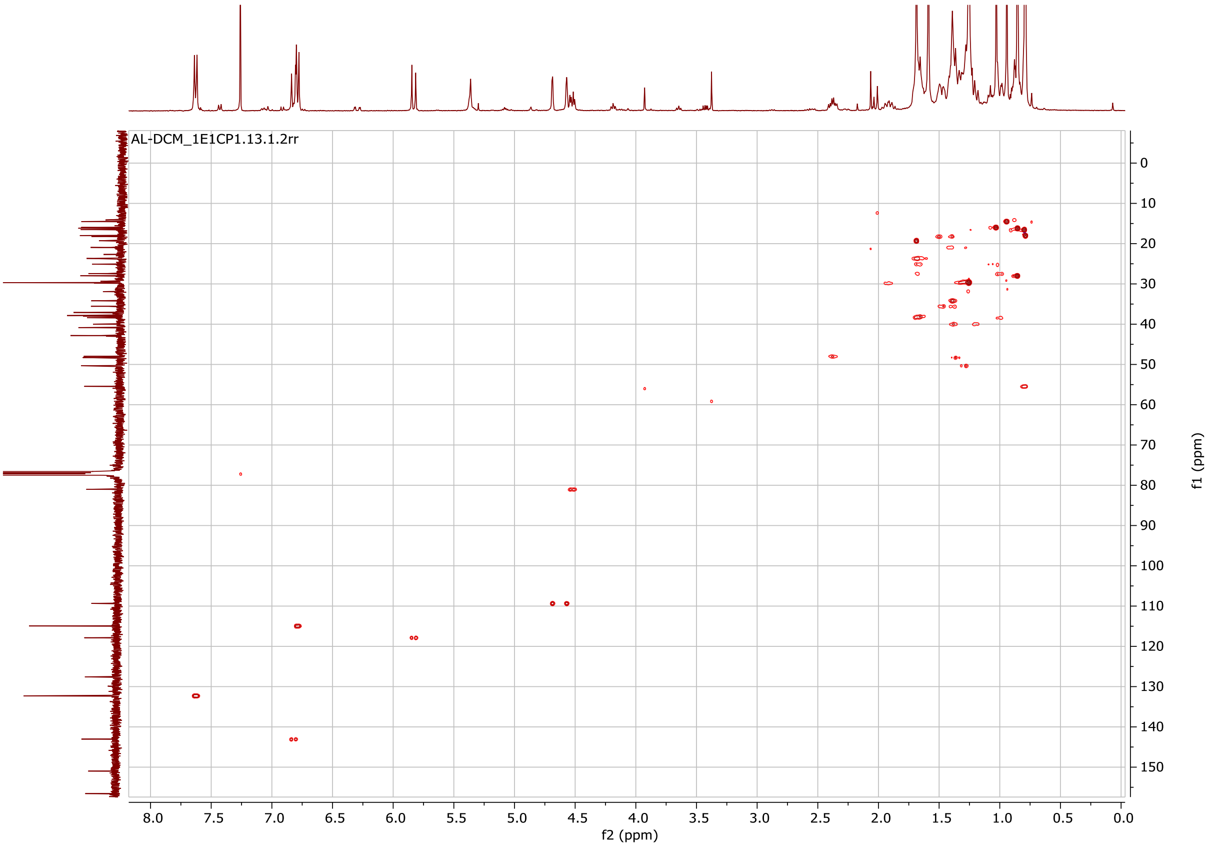

Figure S6 - HMBC spectrum of 3β-*Z*-coumaroyl lupeol **2** (unpurified) ($CDCl_3$)

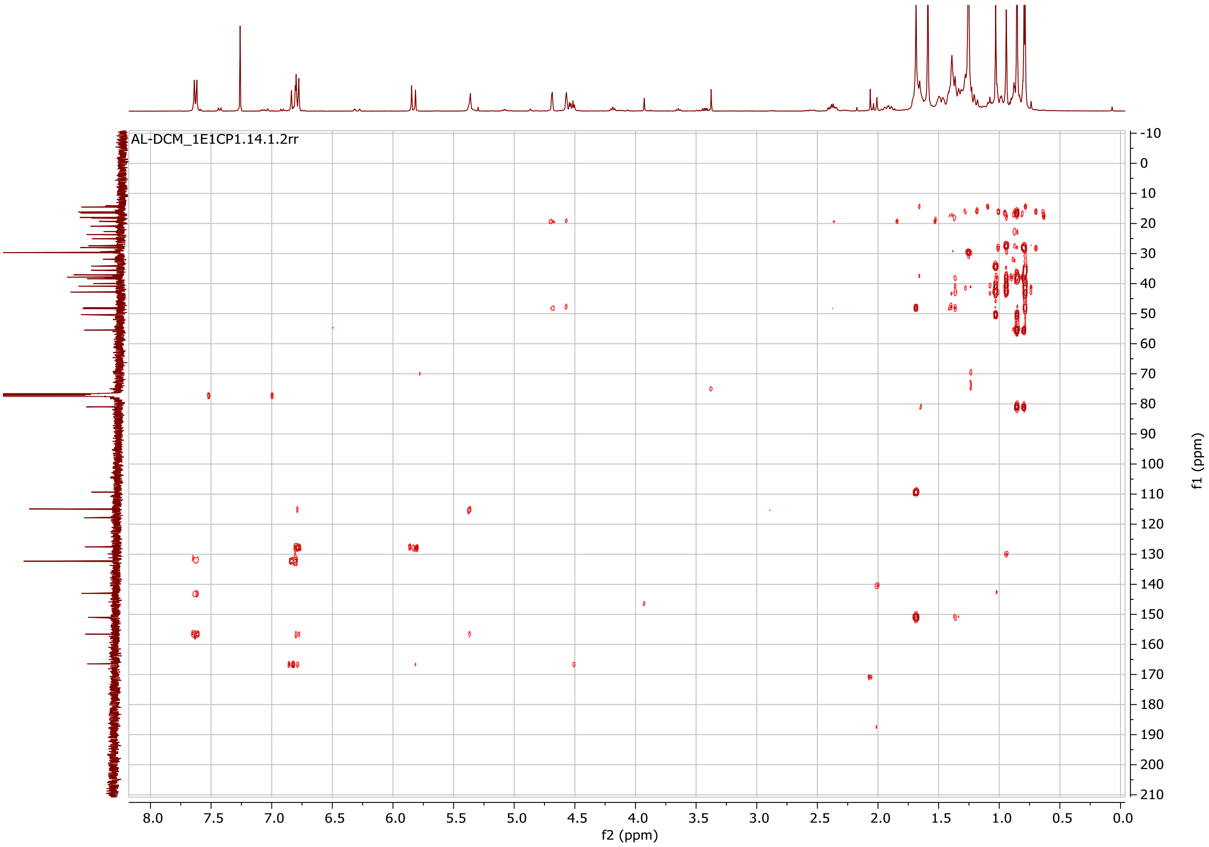

Figure S7 - COSY spectrum of 3β-*Z*-coumaroyl lupeol **2** (unpurified) ($CDCl_3$)

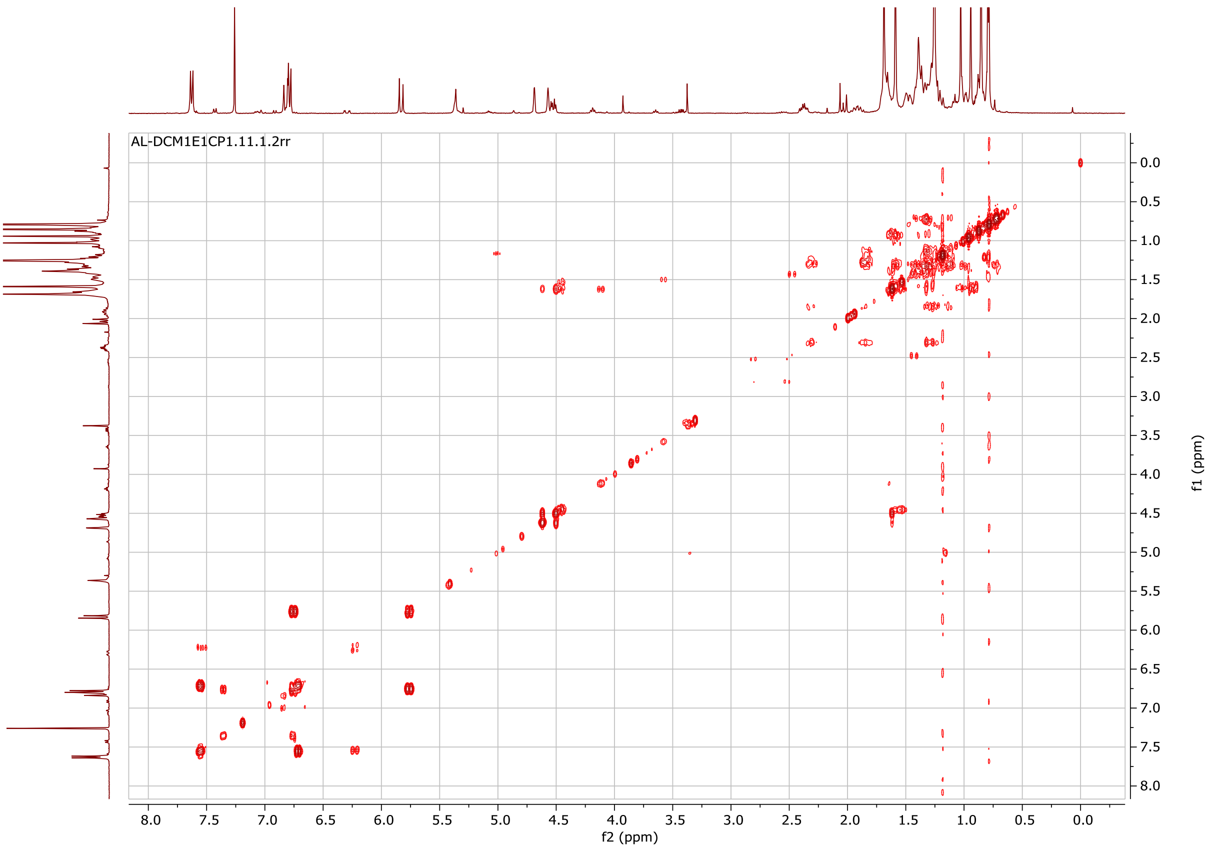

Figure S8 - NOESY spectrum of 3β-*Z*-coumaroyl lupeol **2** (unpurified) ($CDCl_3$)

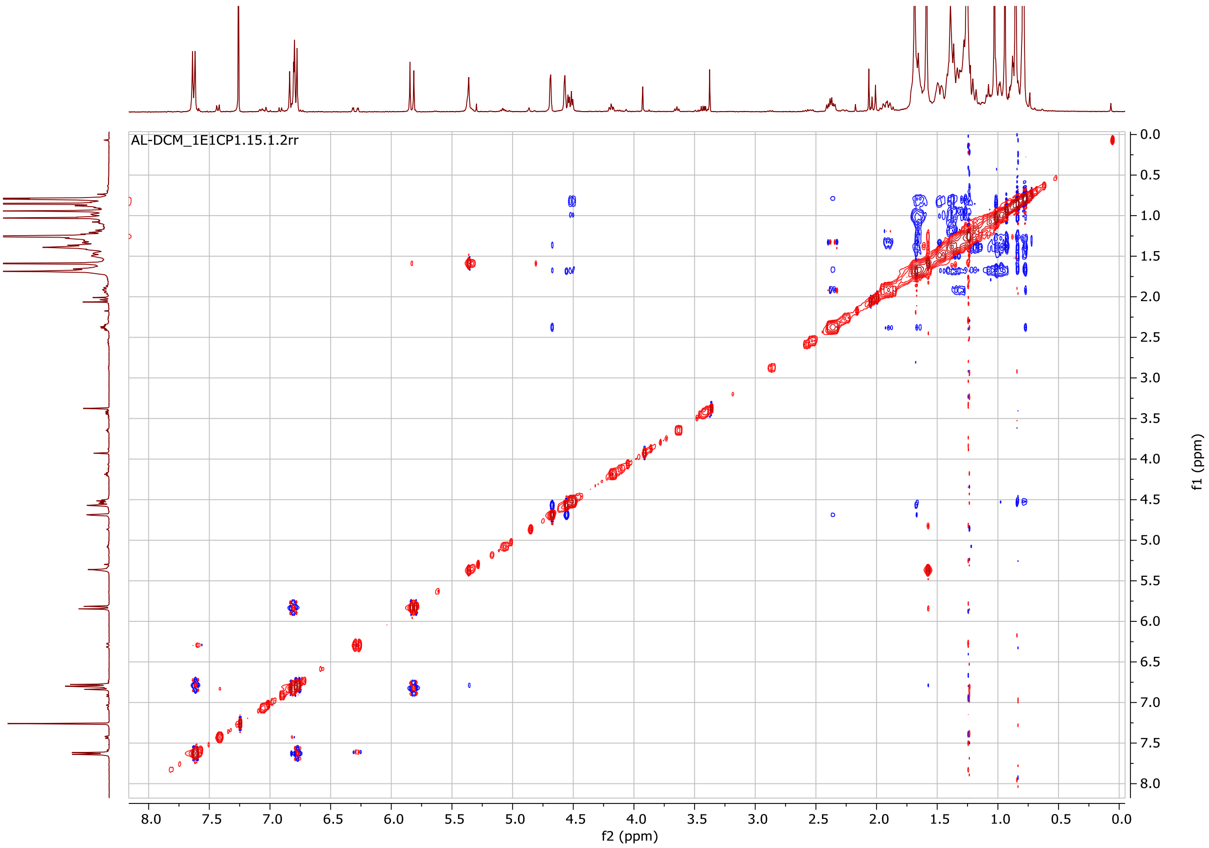

Figure S9 - ^{1}H NMR spectrum of 3β-*E*-coumaroyl lupeol (dioslupecin A) **3** (unpurified) ($CDCl_3$, 400 MHz)

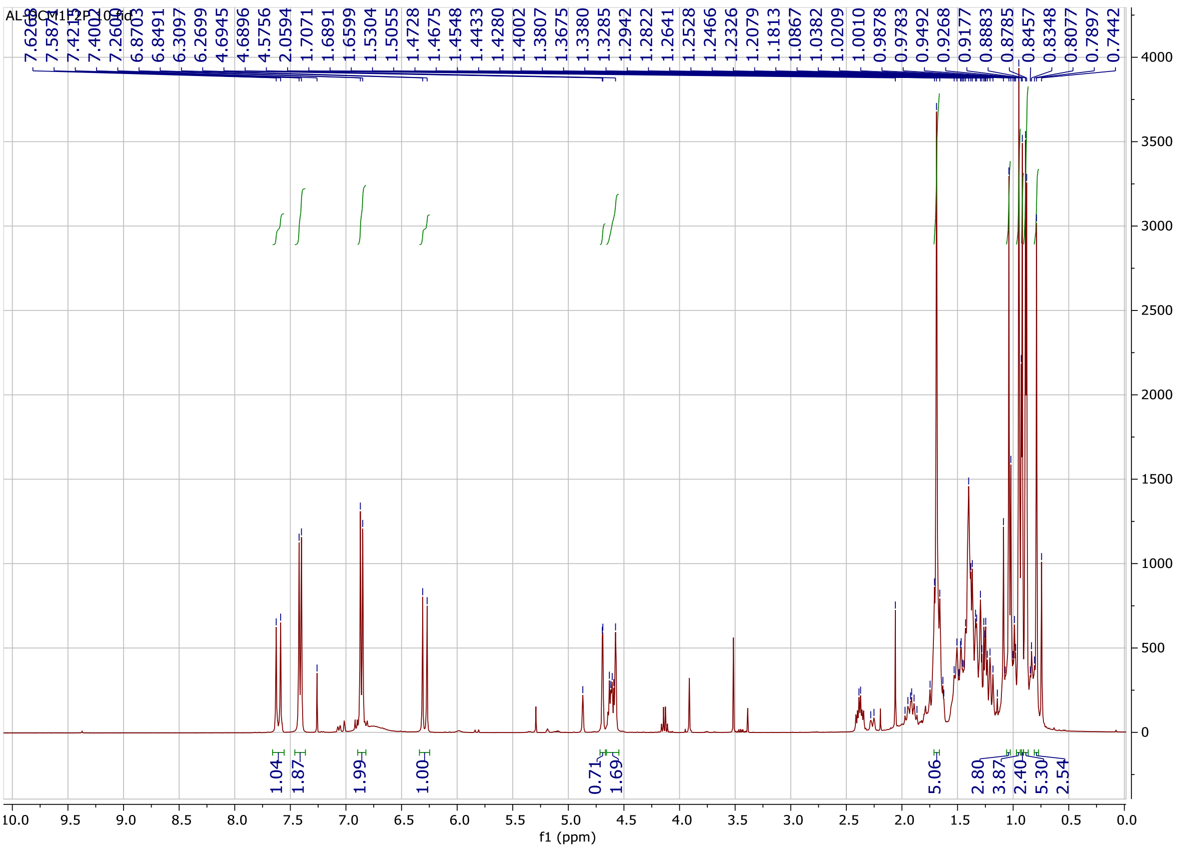

Figure S10 - ^{13}C NMR spectrum of 3β-*E*-coumaroyl lupeol (dioslupecin A) **3** (unpurified) ($CDCl_3$, 100 MHz)

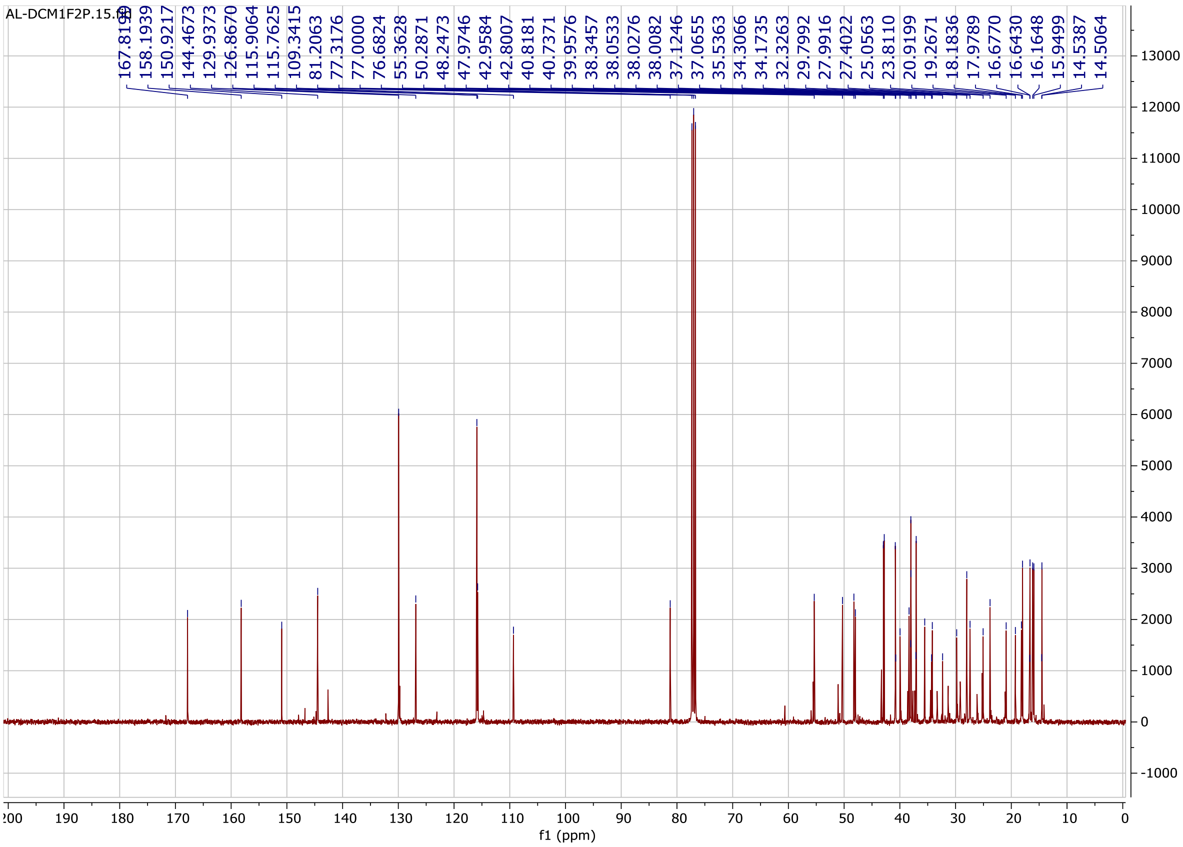

Figure S11 - DEPT135 spectrum of 3β-*E*-coumaroyl lupeol (dioslupecin A) (unpurified) **3**

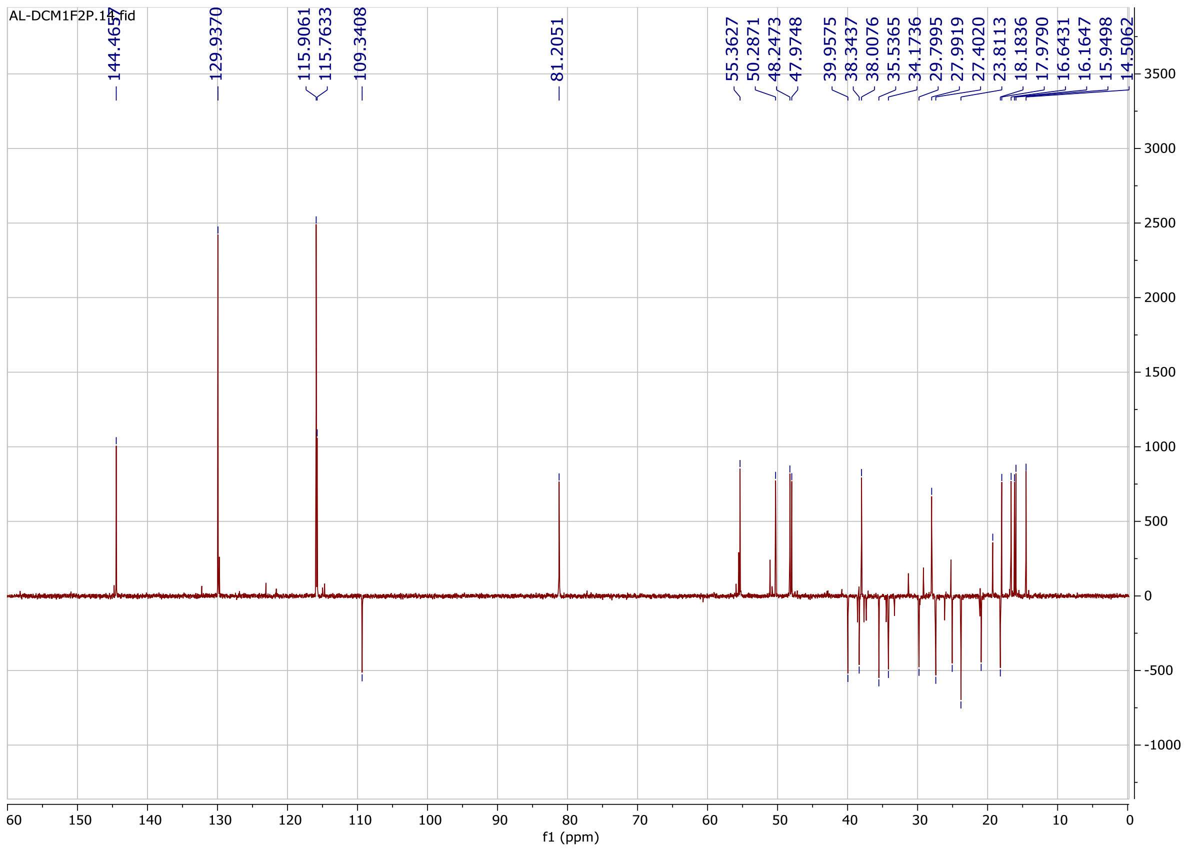

Figure S12 - HSQC spectrum of 3β-*E*-coumaroyl lupeol (dioslupecin A) (unpurified) **3**

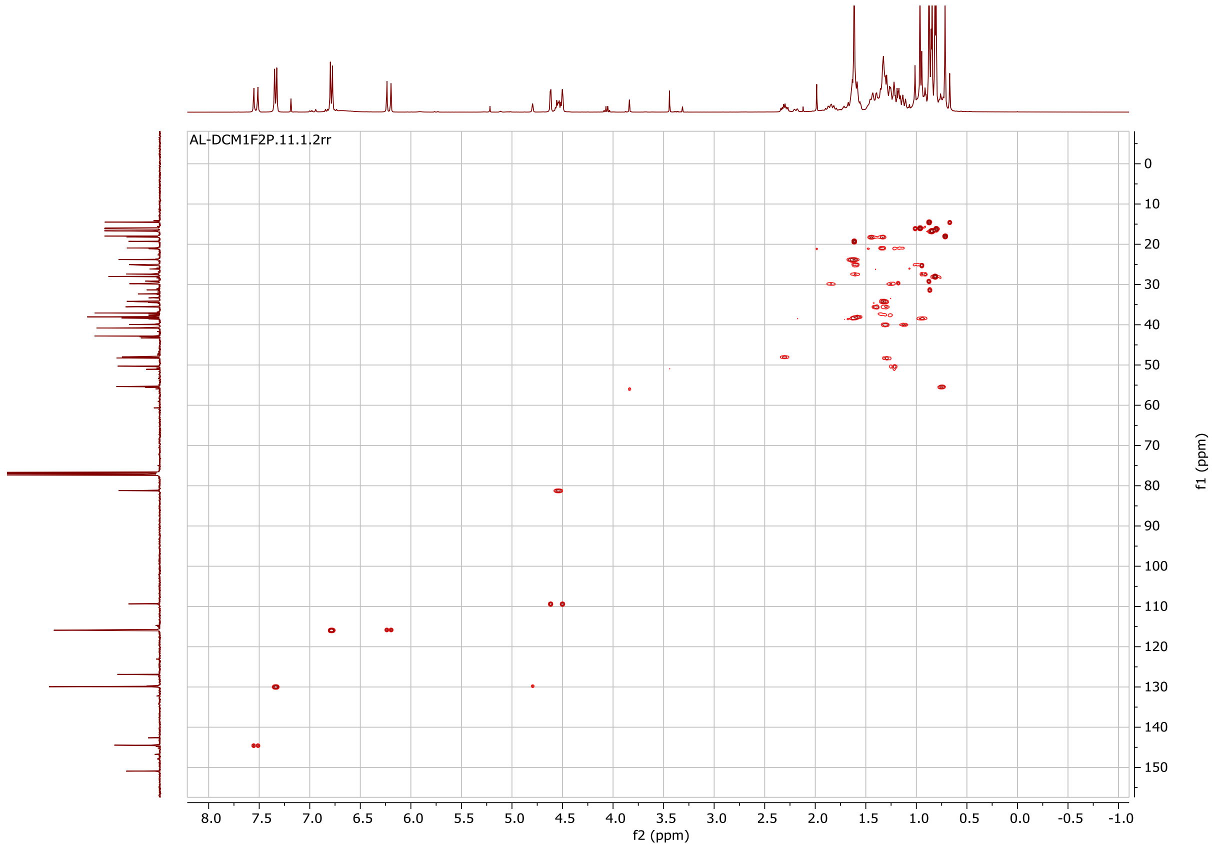

Figure S13 - HMBC spectrum of 3β-*E*-coumaroyl lupeol (dioslupecin A) (unpurified) **3**

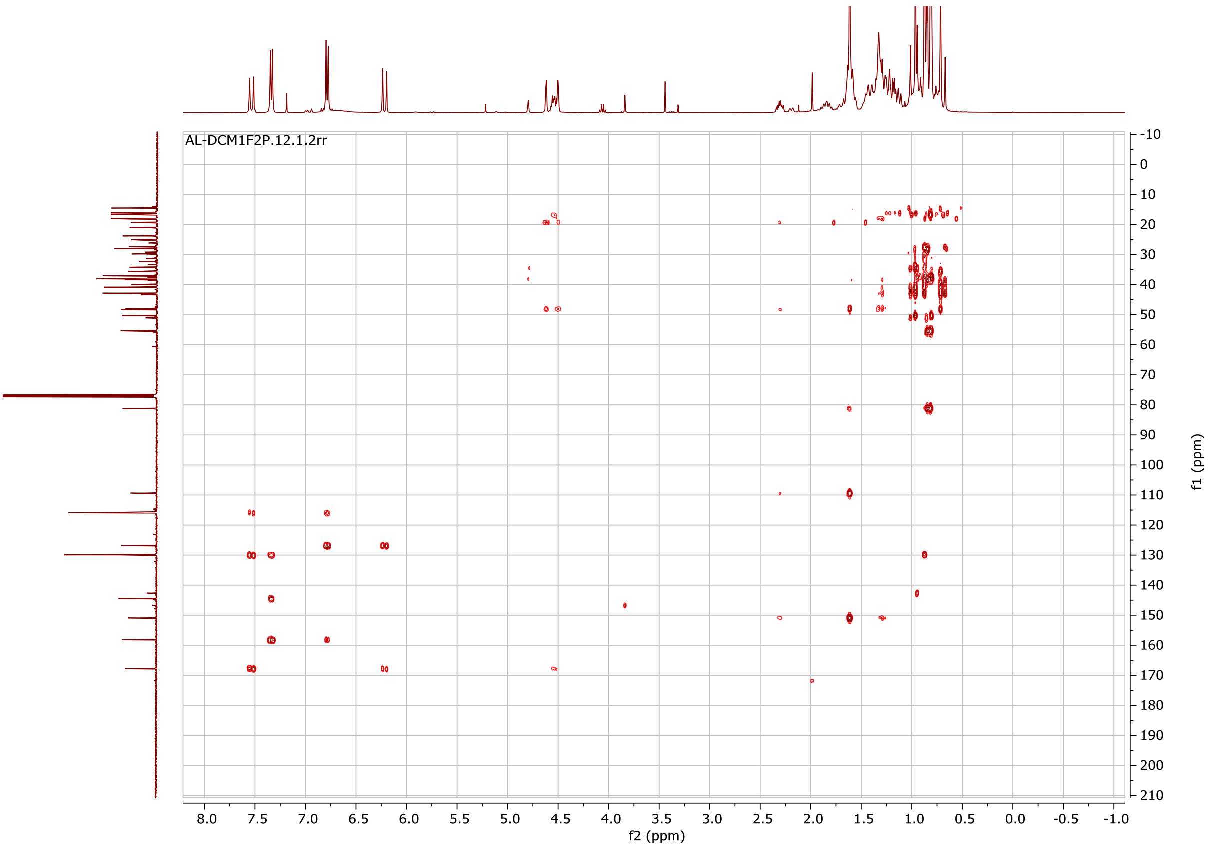

Figure S14 - NOESY spectrum of 3β-*E*-coumaroyl lupeol (dioslupecin A) (unpurified) **3**

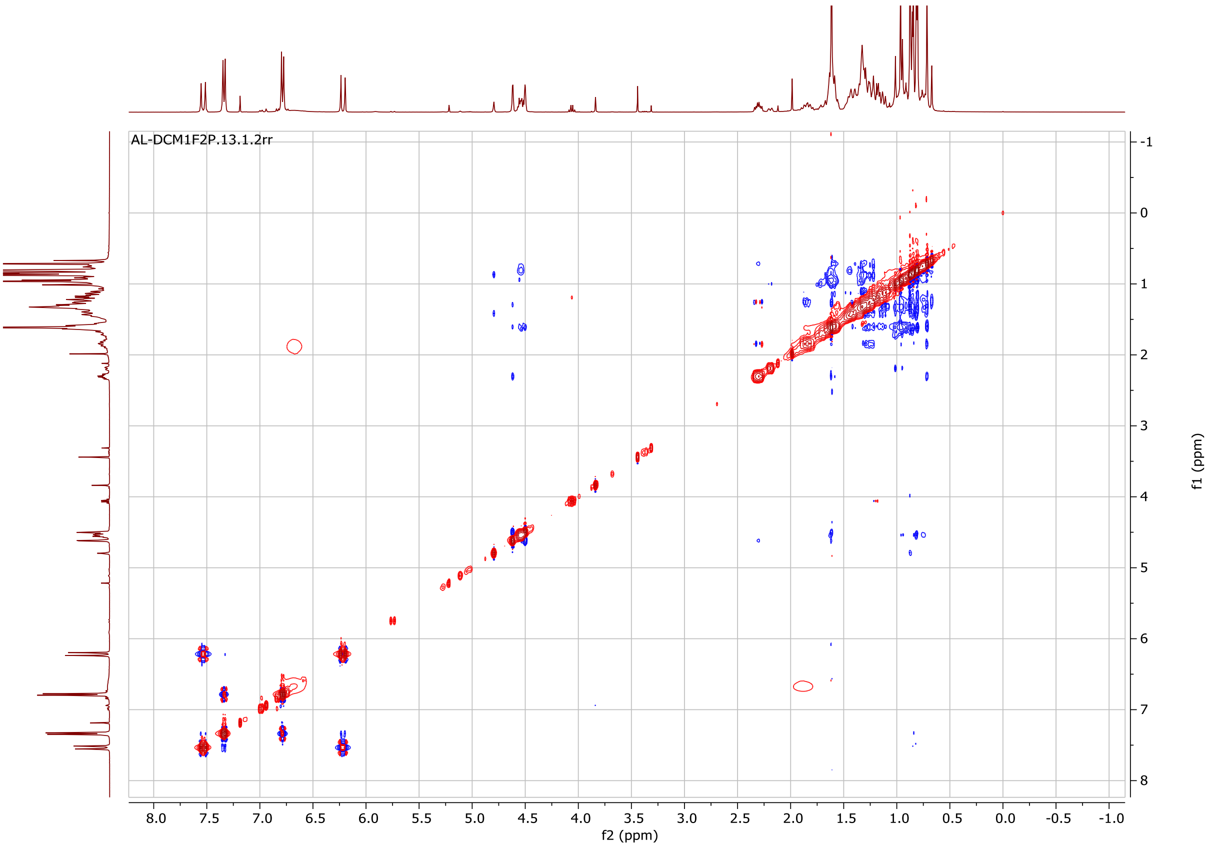

Figure S15 - ^{1}H NMR spectrum of the mixture of 3β-*Z*-coumaroyl lupeol **2** and 3β-*E*-coumaroyl lupeol **3** (1.0:2.0) ($CDCl_3$, 500 MHz)

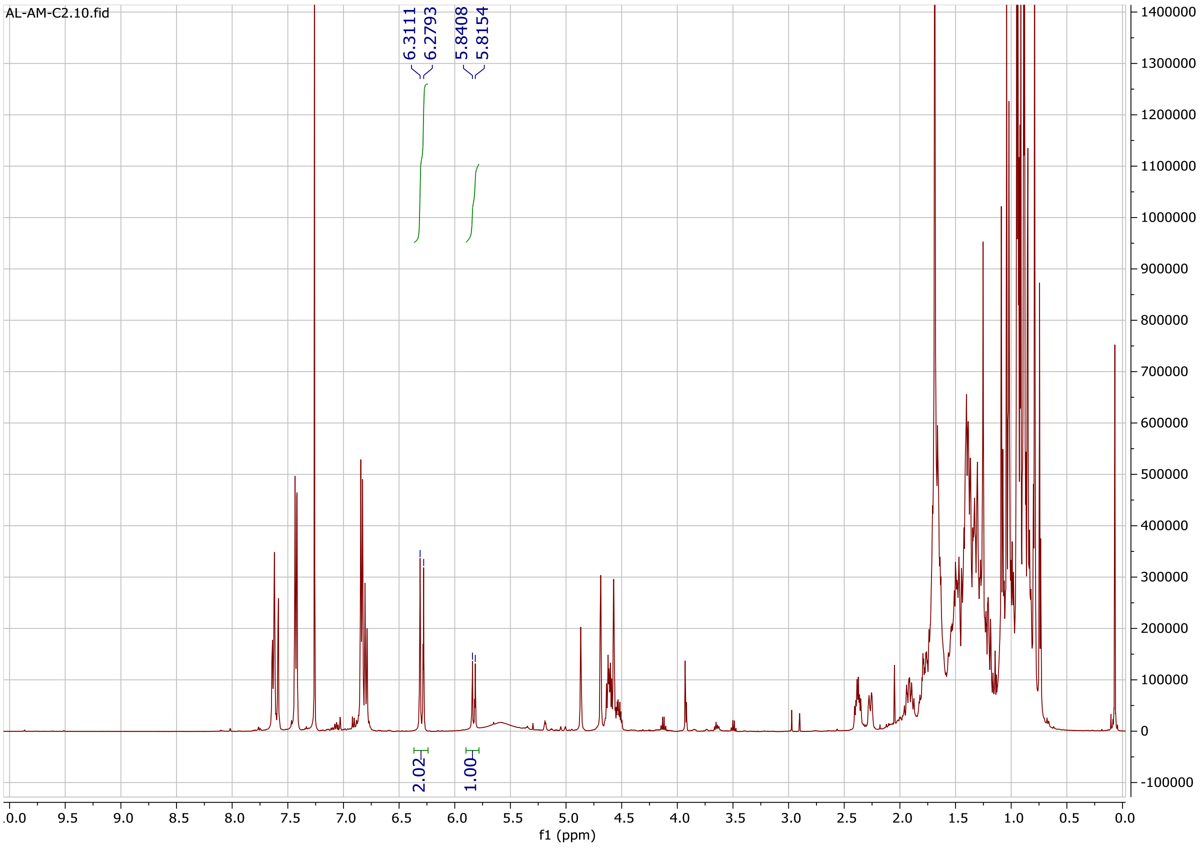

Figure S16 - GC-FID chromatogram of the mixture of 3β-*Z*-coumaroyl lupeol **2** and 3β-*E*-coumaroyl lupeol **3** (1.0:1.6)

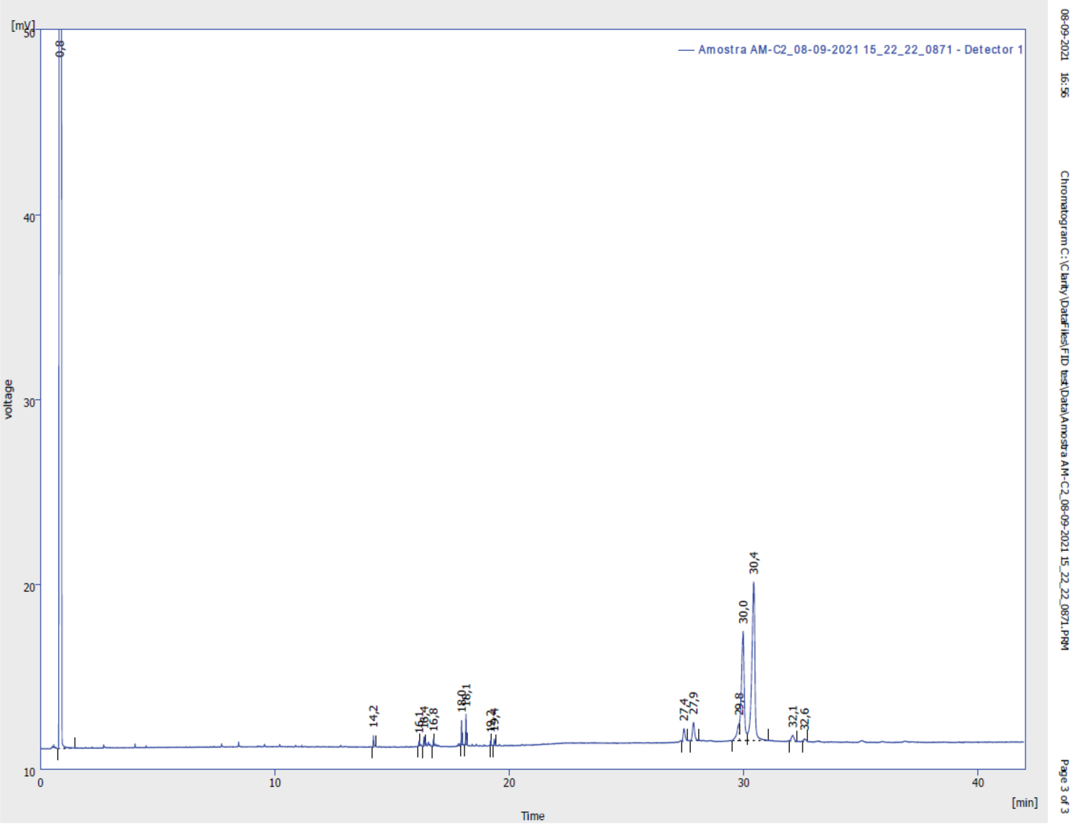

All Signals Result Table (Uncal - Amostra AM-C2_08-09-2021 15_22_22_0871)

	Reten. Time [min]	Area [mV.s]	Height [mV]	Area [%]	Height [%]
1	0,805	69365,755	49262,547	99,77	99,95
2	14,200	1,318	0,615	0,00	0,00
3	16,145	0,541	0,249	0,00	0,00
4	16,362	1,180	0,487	0,00	0,00
5	16,758	0,709	0,372	0,00	0,00
6	17,958	2,398	1,301	0,00	0,00
7	18,148	3,096	1,645	0,00	0,00
8	19,192	0,478	0,255	0,00	0,00
9	19,358	0,907	0,366	0,00	0,00
10	27,445	3,672	0,677	0,01	0,00
11	27,855	6,273	0,987	0,01	0,00
12	29,802	6,245	0,919	0,01	0,00
13	29,970	50,427	5,903	0,07	0,01
14	30,420	79,262	8,577	0,11	0,02
15	32,090	2,551	0,323	0,00	0,00
16	32,590	0,960	0,143	0,00	0,00
	All Signals Total	69525,772	49285,366	100,00	100,00

Chromatogram Comments - Amostra AM-C2_08-09-2021 15_22_22_0871

Figure S17 - ^{1}H NMR spectrum of the mixture of 3β-*Z*-coumaroyl lupeol **2** and 3β-*E*-coumaroyl lupeol **3** (1.0:4.4) (CDCl$_3$, 500 MHz)

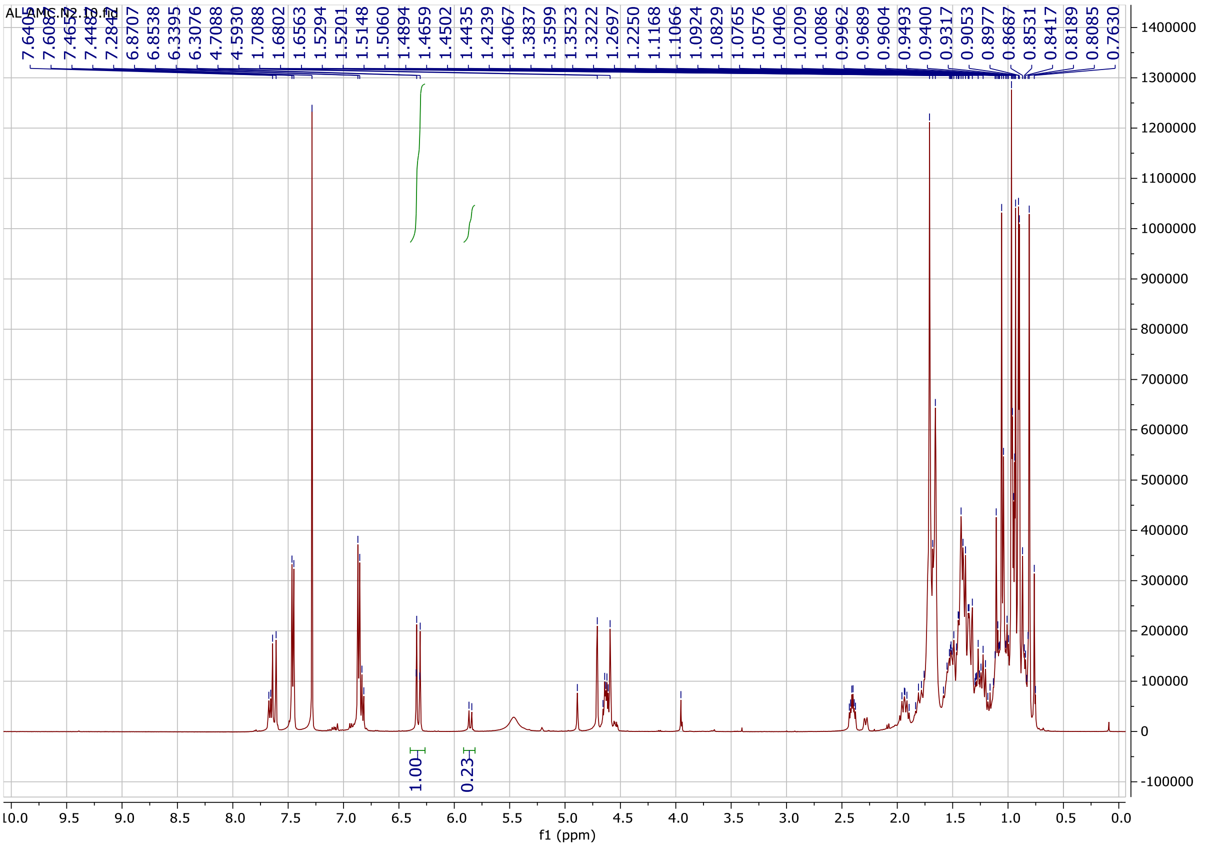

Figure S18 - ^{1}H NMR spectrum of kolavic acid 15-methyl ester **4** (CDCl$_3$, 500 MHz)

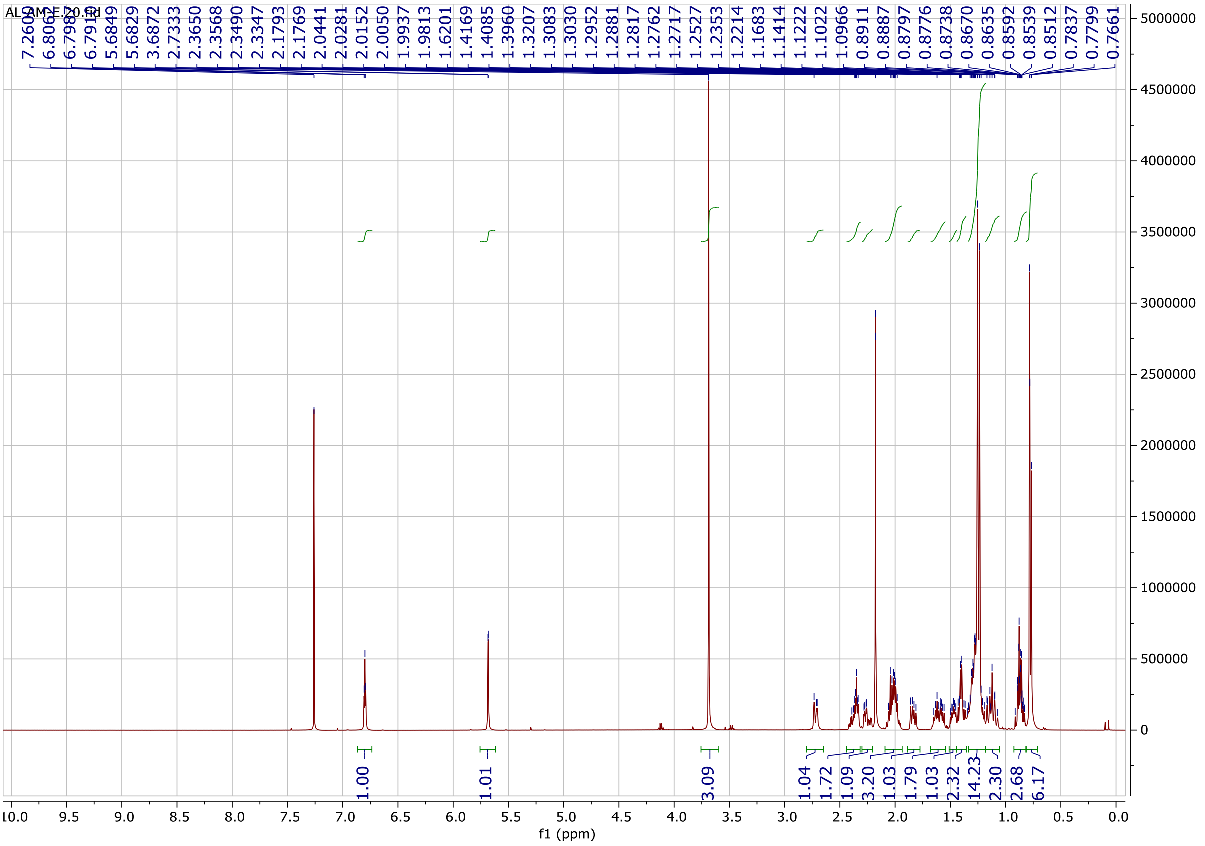

Figure S19 - ^{13}C NMR spectrum of kolavic acid 15-methyl ester **4** ($CDCl_3$, 125 MHz)

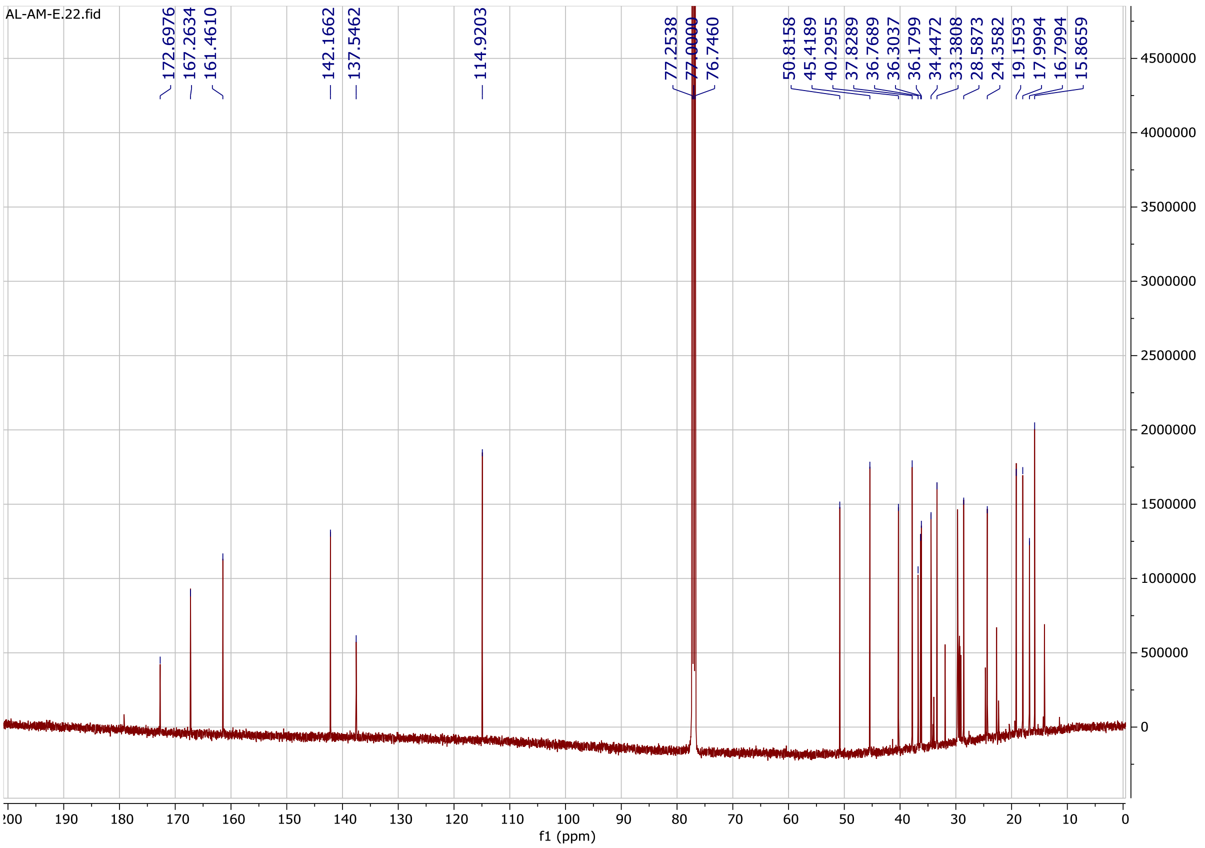

Figure S20 - DEPT135 spectrum of kolavic acid 15-methyl ester **4** ($CDCl_3$)

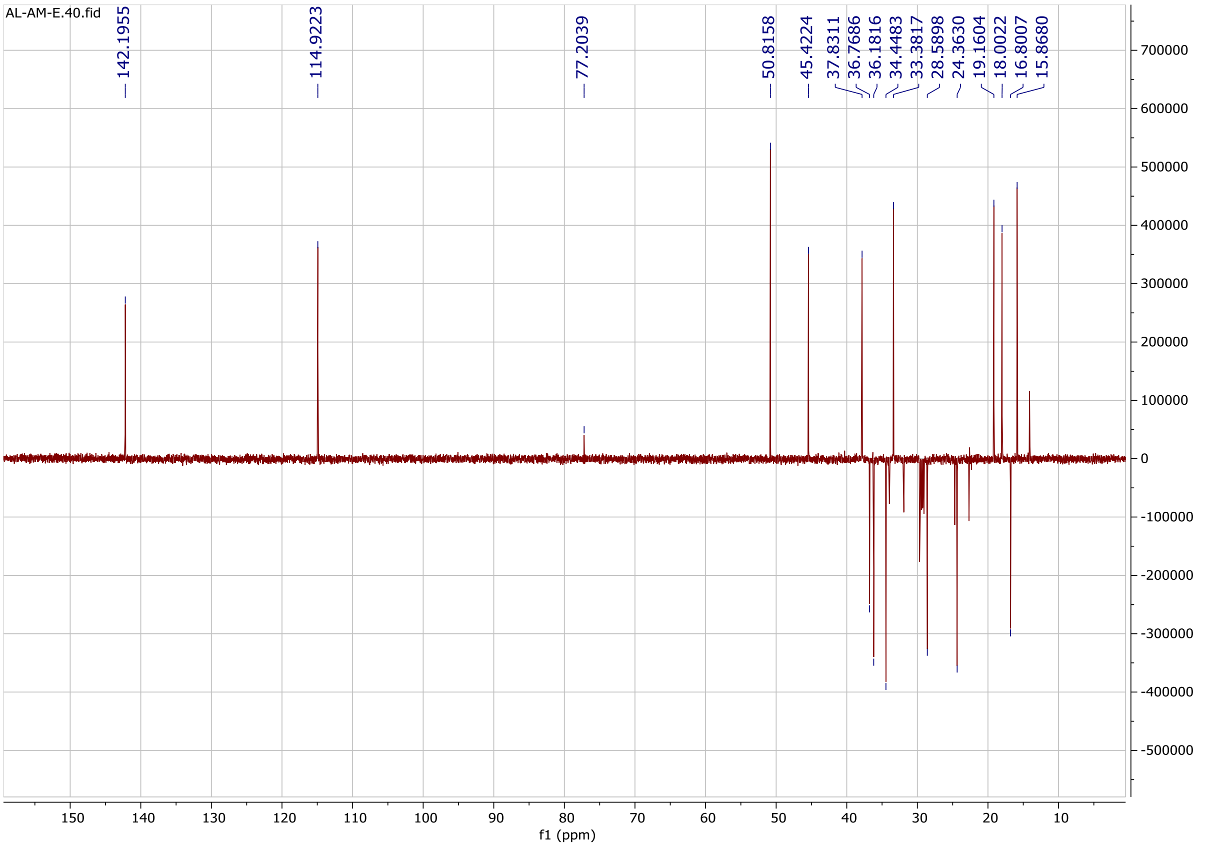

Figure S21 - HSQC spectrum of kolavic acid 15-methyl ester **4** ($CDCl_3$)

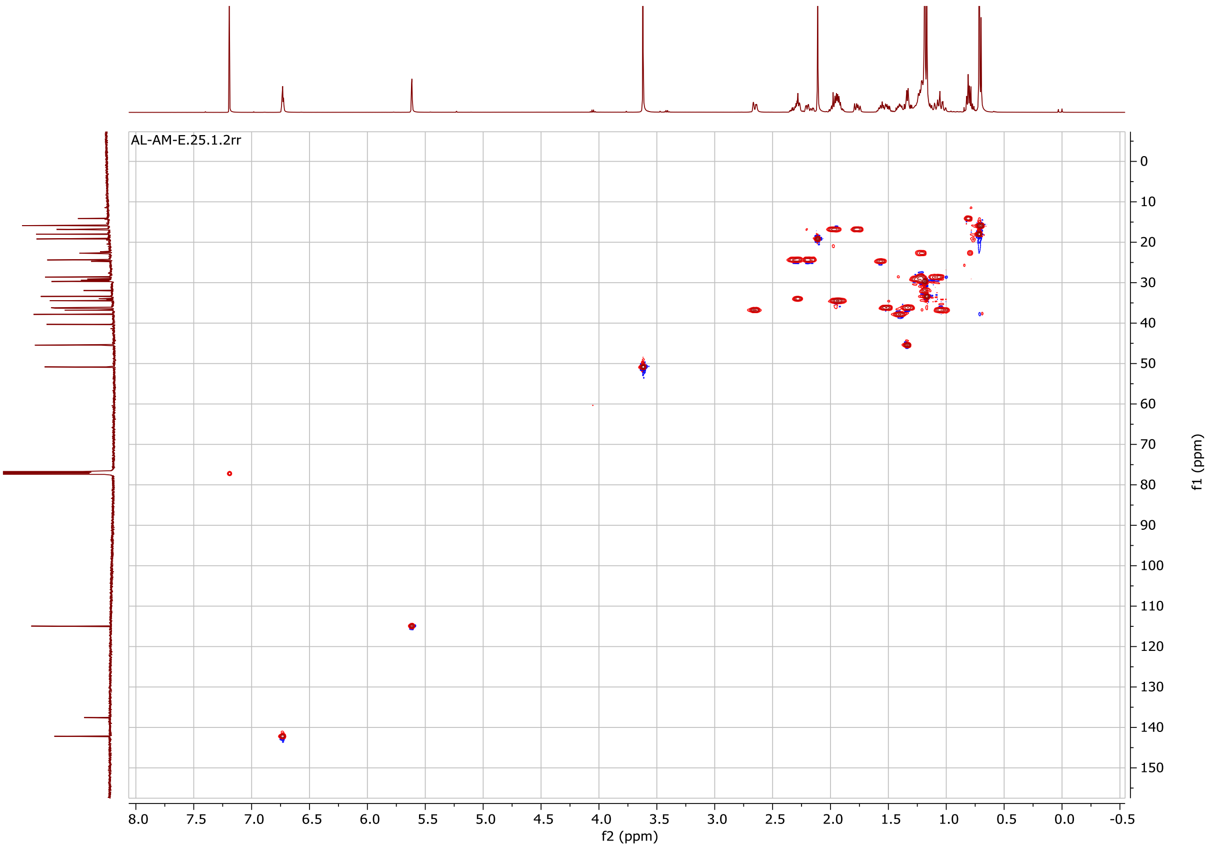

Figure S22 - HMBC spectrum of kolavic acid 15-methyl ester **4** ($CDCl_3$)

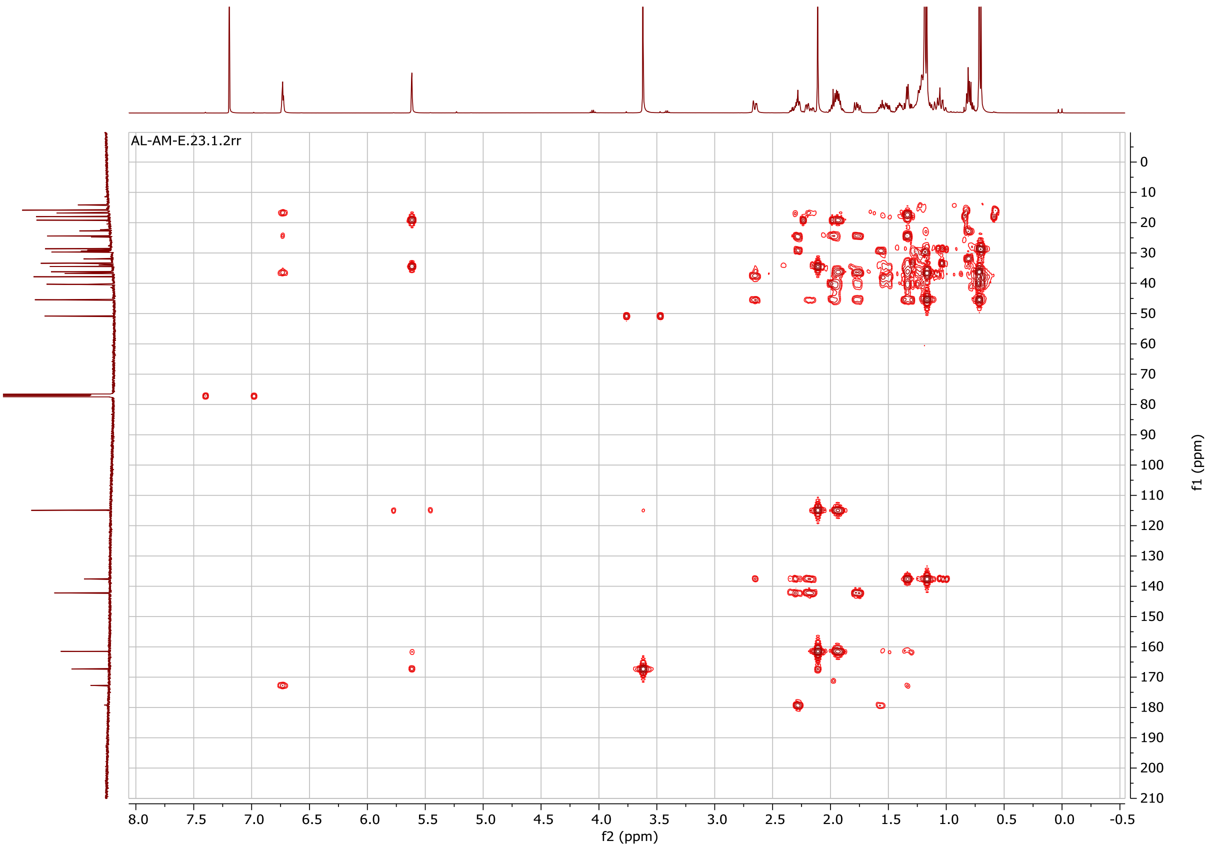

Figure S23 - COSY spectrum of kolavic acid 15-methyl ester **4** ($CDCl_3$)

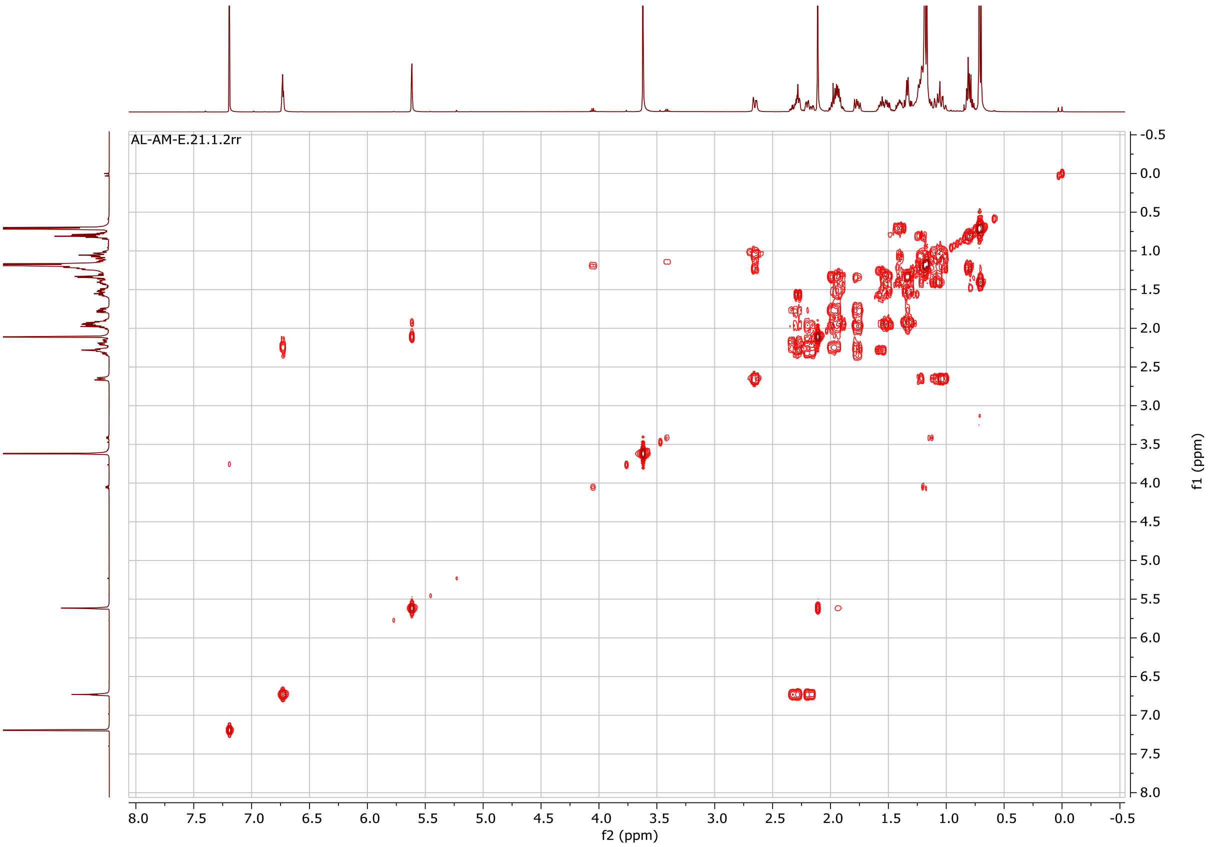

Figure S24 - NOESY spectrum of kolavic acid 15-methyl ester **4** ($CDCl_3$)

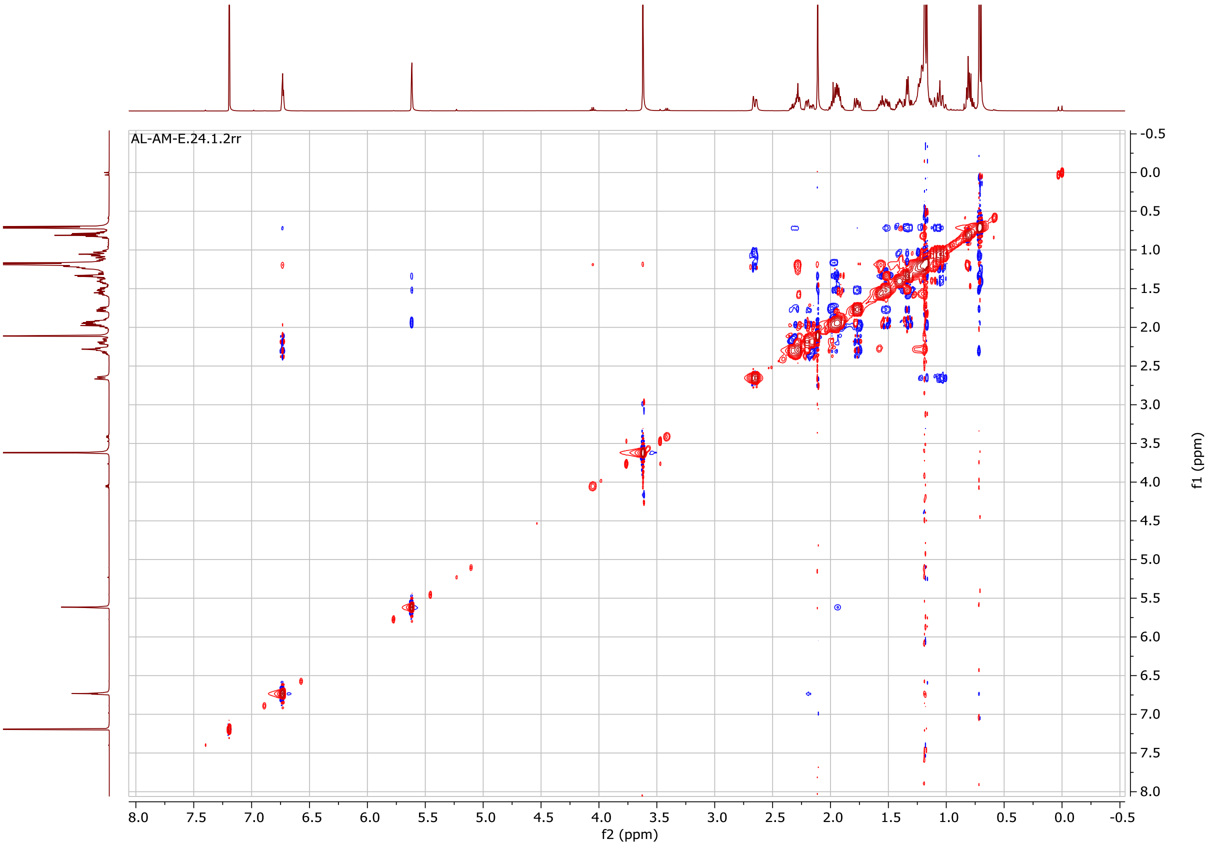

Figure S25 - ESI-MS spectra of kolavic acid 15-methyl ester **4**

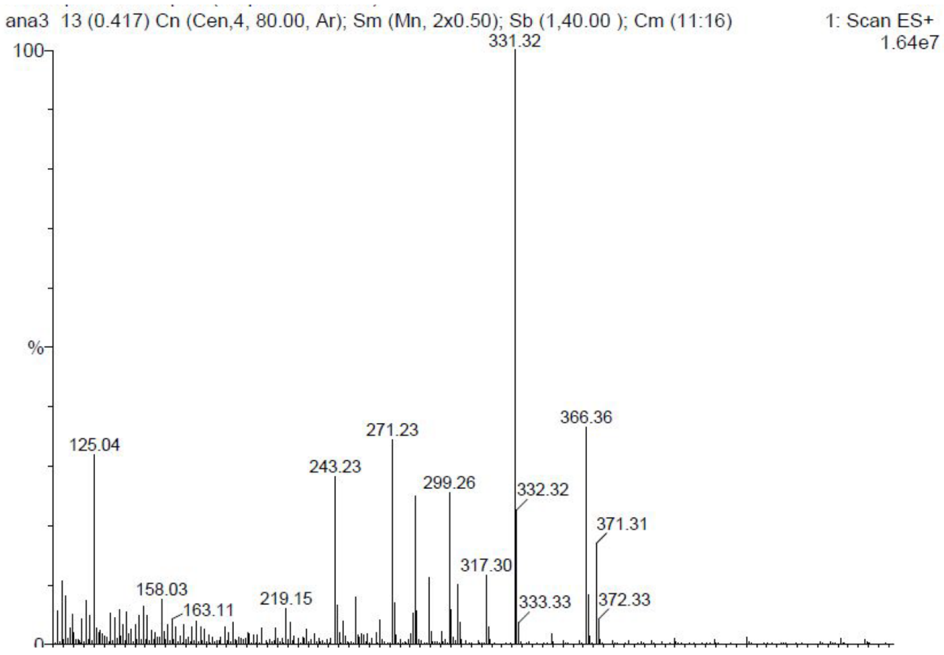

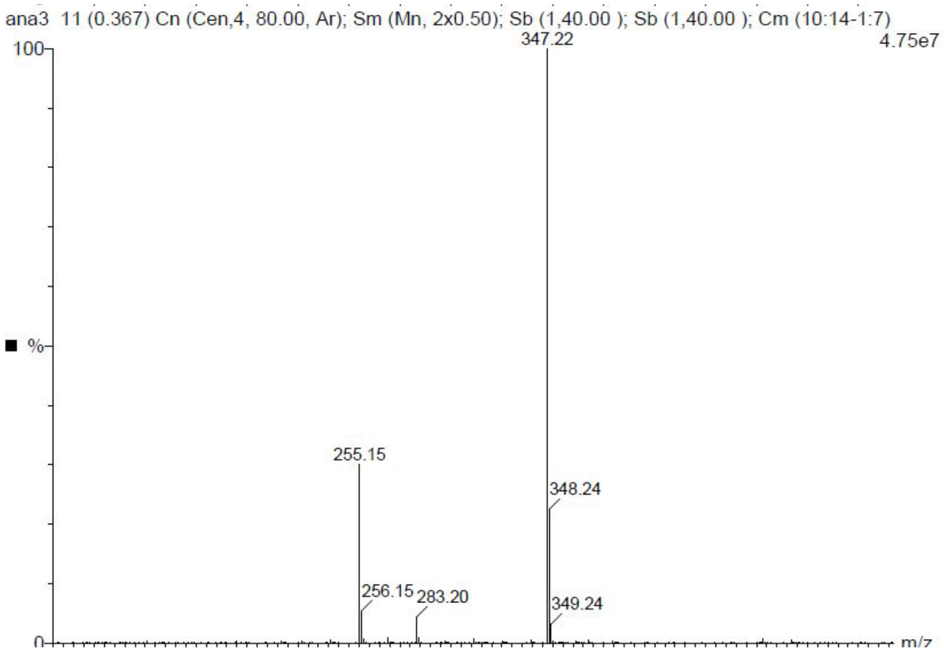

Figure S26 - ^{1}H NMR spectrum of vomifoliol (blumenol A) **5** (unpurified) ($CDCl_3$, 500 MHz)

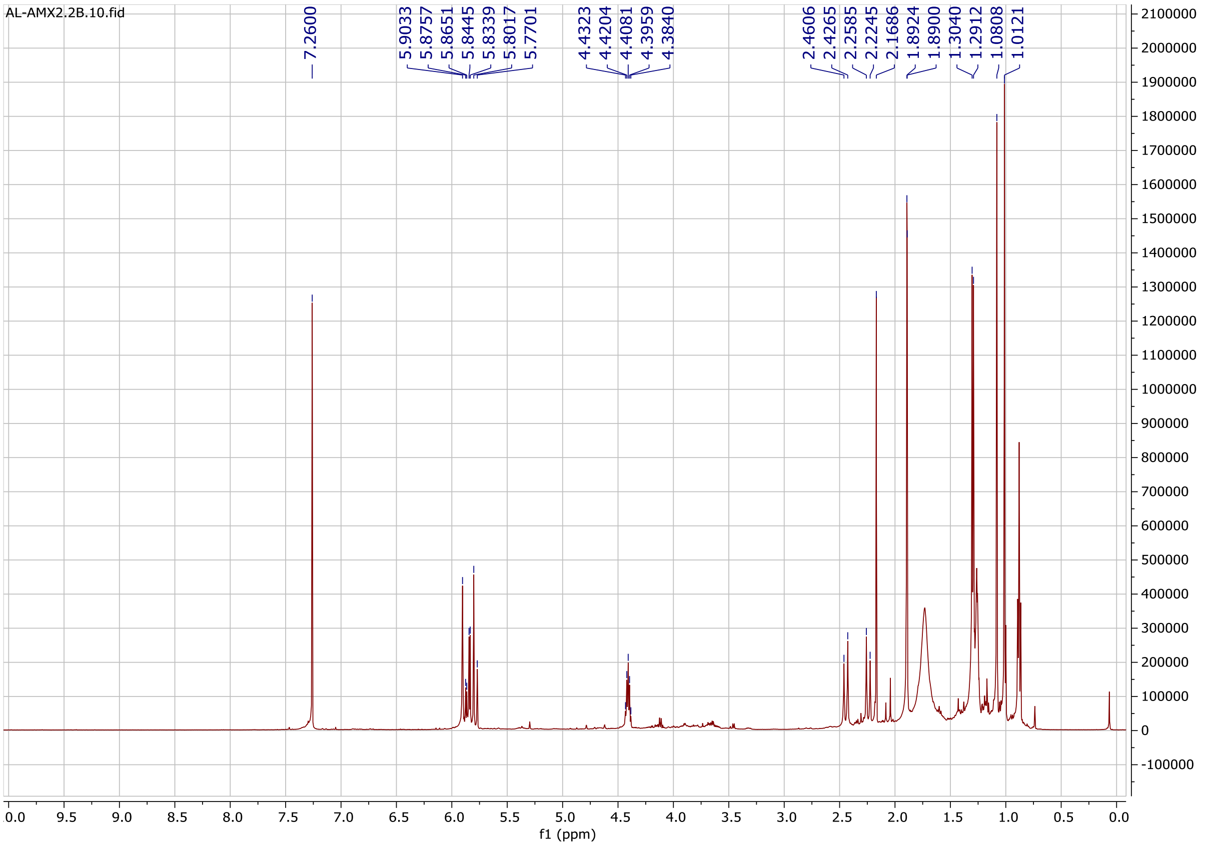

Figure S27 - ^{13}C NMR spectrum of vomifoliol (blumenol A) **5** (unpurified) ($CDCl_3$, 125 MHz)

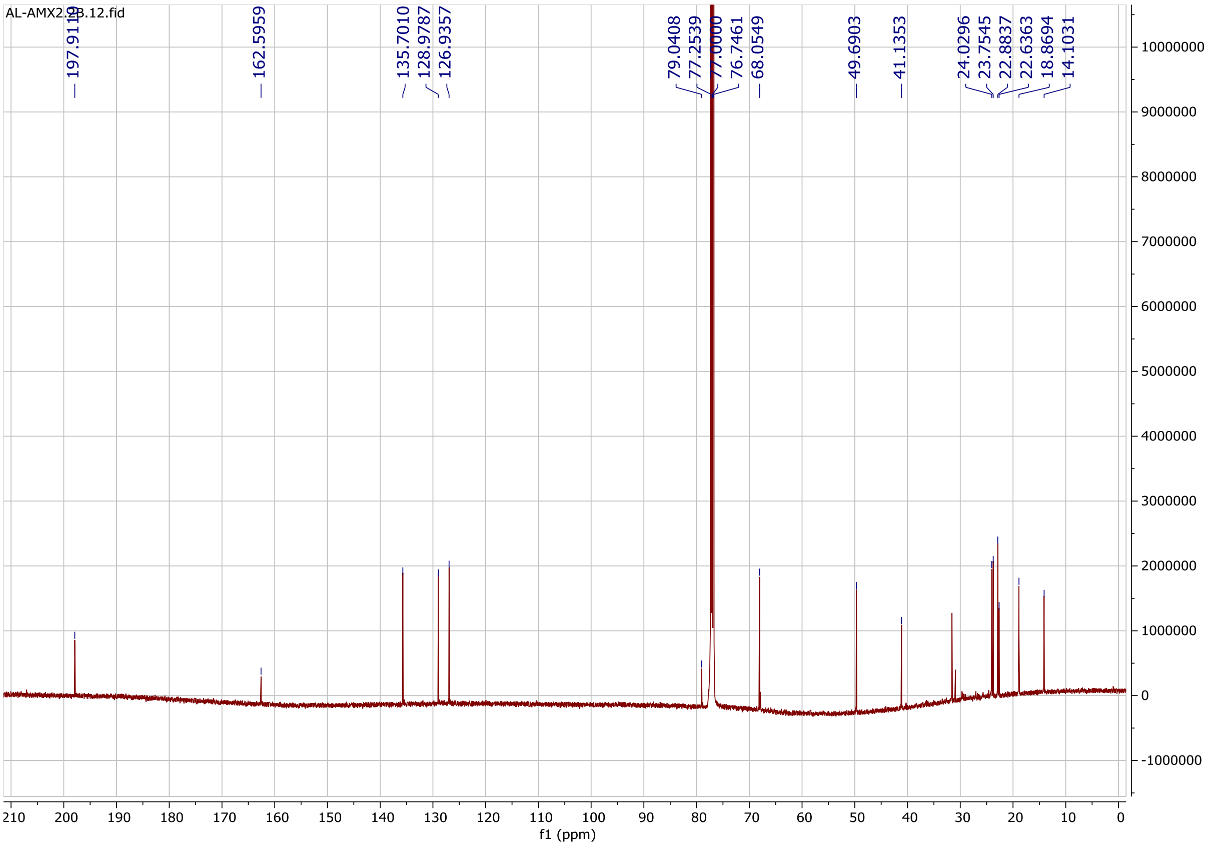

Figure S28 - DEPT135 spectrum of vomifoliol (blumenol A) **5** (unpurified) ($CDCl_3$)

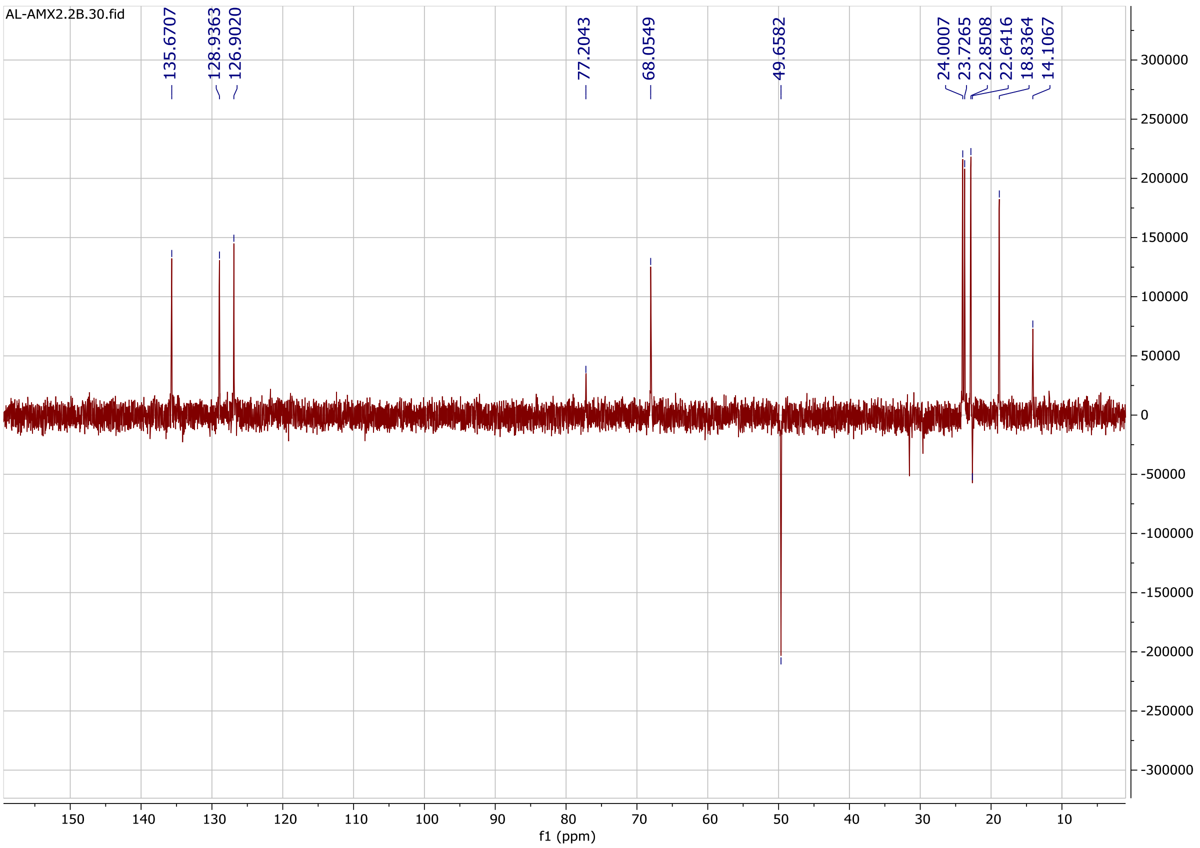

Figure S29 - HSQC spectrum of vomifoliol (blumenol A) **5** (unpurified) ($CDCl_3$)

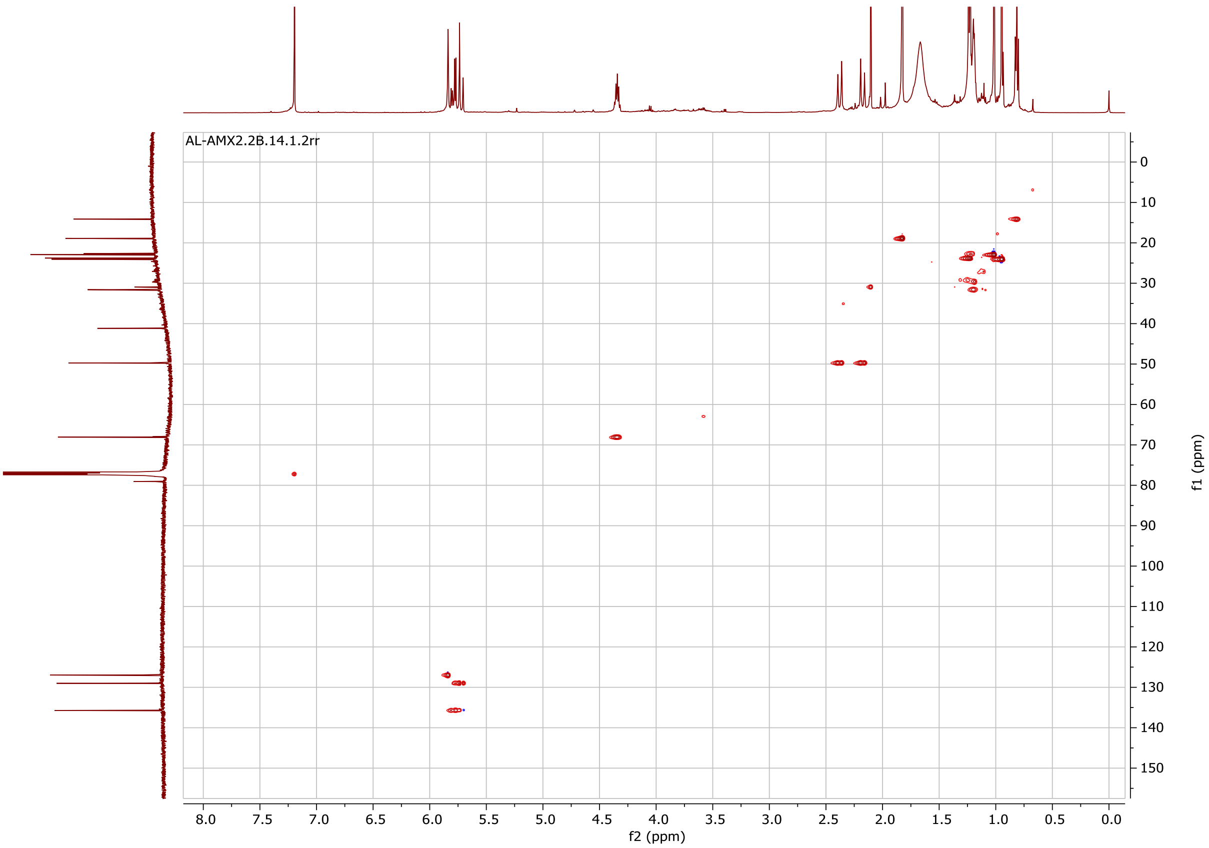

Figure S30 - HMBC spectrum of vomifoliol (blumenol A) **5** (unpurified) ($CDCl_3$)

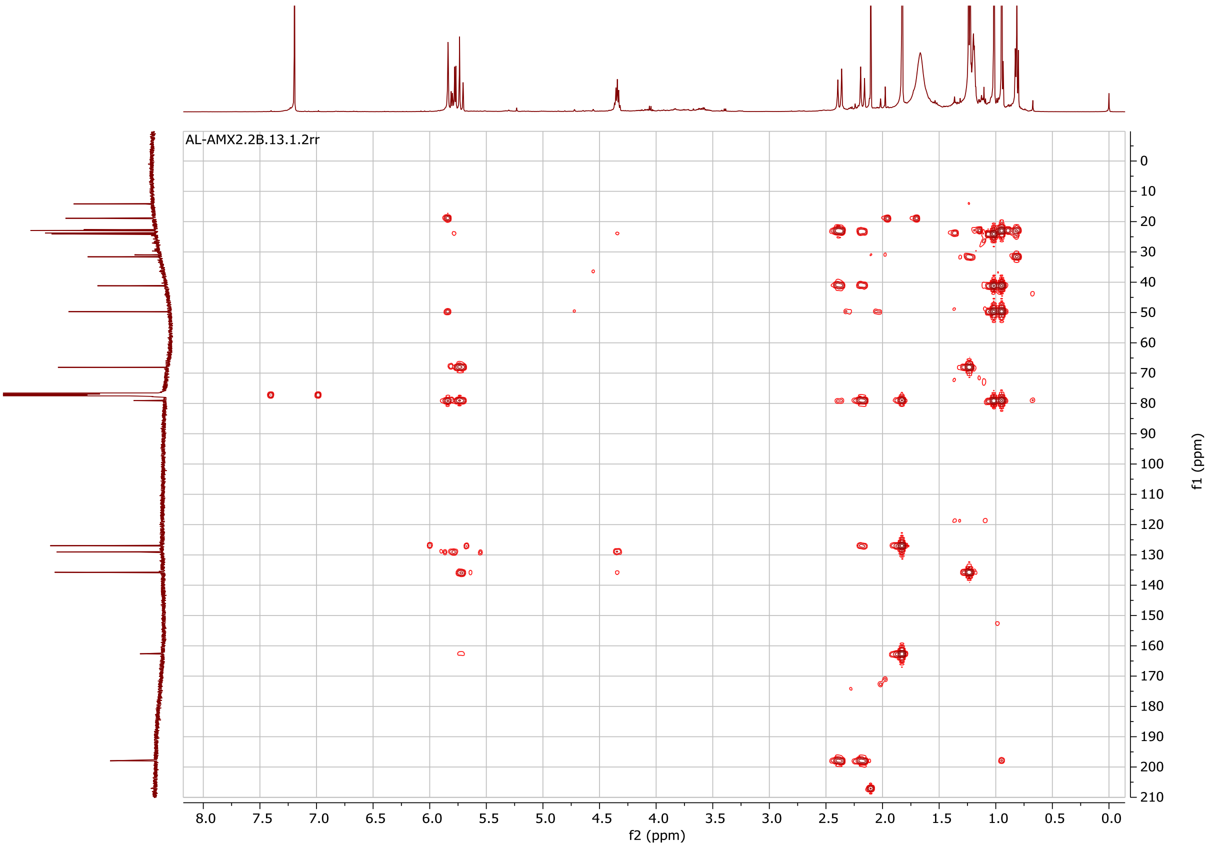

Figure S31 - COSY spectrum of vomifoliol (blumenol A) **5** (unpurified) ($CDCl_3$)

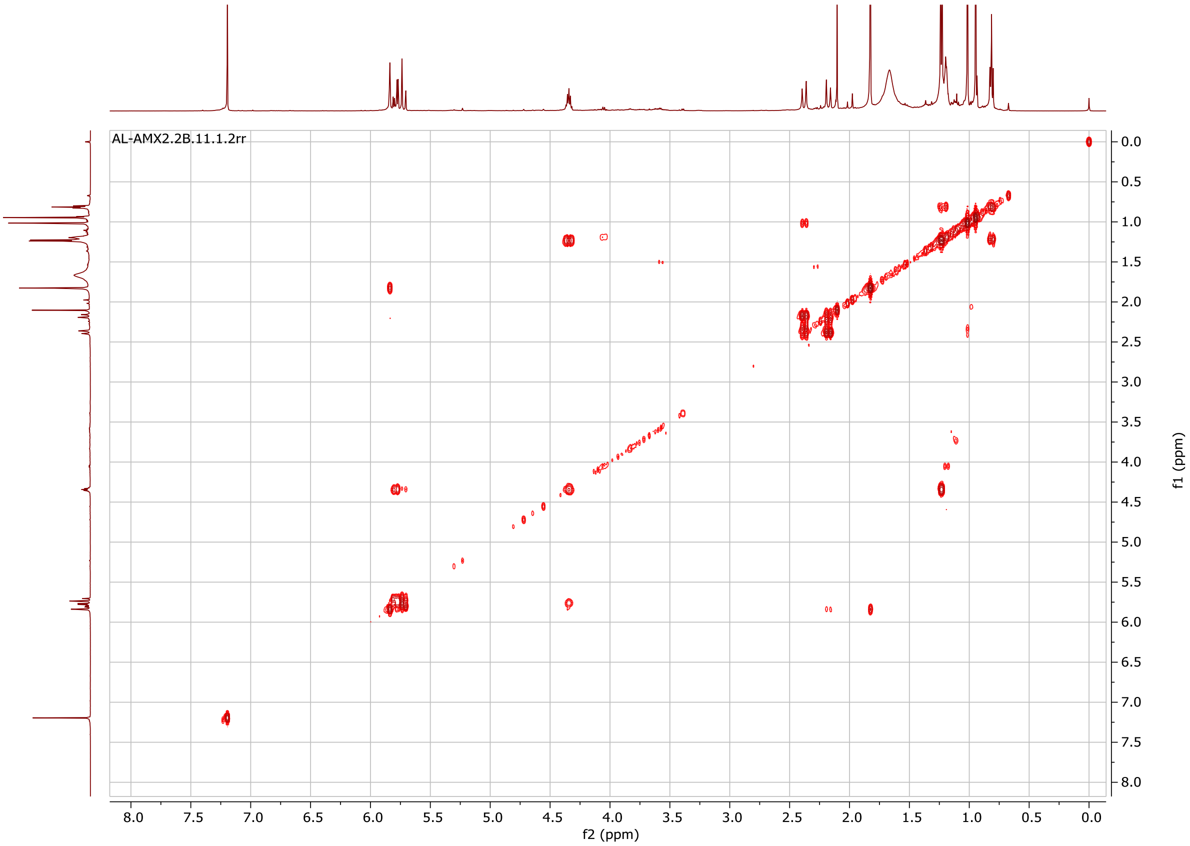

Figure S32 - ESI-MS spectrum of vomifoliol (blumenol A) **5** (unpurified)

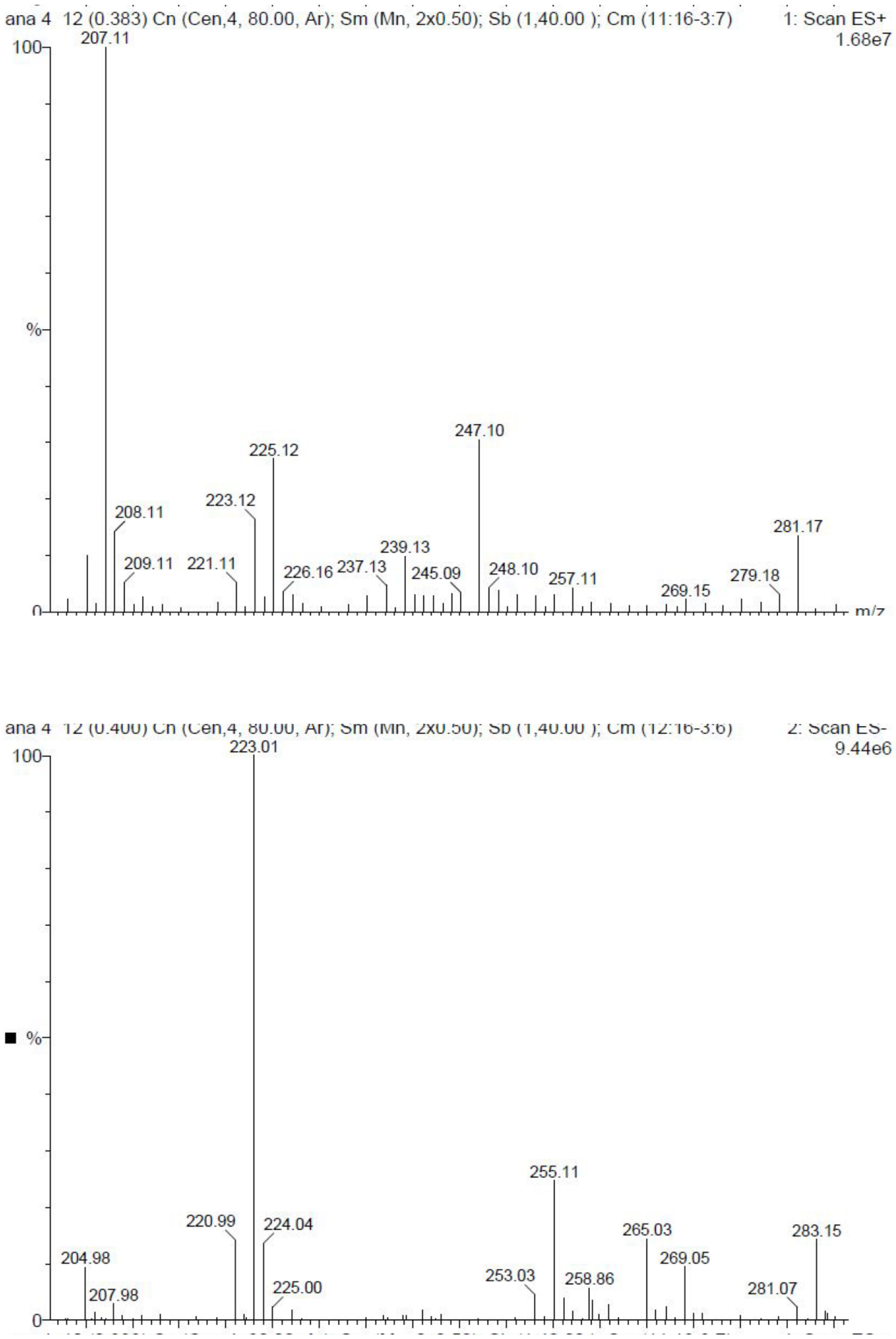

Supplement: Supplementary file 1 [file plants-10-02698-s001.zip › plants-1484467-supplementary.pdf]
